# Evaluation of Lesions and Viral Antigen Distribution in Domestic Pigs Inoculated Intranasally with African Swine Fever Virus Ken05/Tk1 (Genotype X)

**DOI:** 10.3390/pathogens10060768

**Published:** 2021-06-18

**Authors:** Pedro J. Sánchez-Cordón, Tobias Floyd, Daniel Hicks, Helen R. Crooke, Stephen McCleary, Ronan R. McCarthy, Rebecca Strong, Linda K. Dixon, Aleksija Neimanis, Emil Wikström-Lassa, Dolores Gavier-Widén, Alejandro Núñez

**Affiliations:** 1 Pathology and Animal Sciences Department, Animal and Plant Health Agency (APHA-Weybridge), Woodham Lane, New Haw, Addlestone KT15 3NB, UK; tobias.floyd@apha.gov.uk (T.F.); daniel.hicks@apha.gov.uk (D.H.); 2 Pathology Department, Centro de Investigación en Sanidad Animal (CISA), Instituto Nacional de Investigación y Tecnología Agraria y Alimentaria (INIA-CSIC), Carretera Algete-El Casar Km. 8.1, Valdeolmos, 28130 Madrid, Spain; 3 Virology Department, Animal and Plant Health Agency (APHA-Weybridge), Woodham Lane, New Haw, Addlestone KT15 3NB, UK; helen.crooke@apha.gov.uk (H.R.C.); stephen.mccleary@apha.gov.uk (S.M.); Ronan.Mccarthy@brunel.ac.uk (R.R.M.); rebecca.strong@apha.gov.uk (R.S.); 4 The Pirbright Institute, Ash Road, Pirbright, Woking, Surrey GU24 0NF, UK; linda.dixon@pirbright.ac.uk; 5 Department of Pathology and Wildlife Diseases, National Veterinary Institute (SVA), 751 89 Uppsala, Sweden; aleksija.neimane@sva.se (A.N.); emil.wikstrom@sva.se (E.W.-L.); dolores.gavier-widen@sva.se (D.G.-W.); 6 Department of Biomedical Sciences and Veterinary Public Health, Swedish University of Agricultural Sciences (SLU), Box 7028, 750 07 Uppsala, Sweden

**Keywords:** African swine fever virus, genotype X, domestic pig, *Sus scrofa domesticus*, infection routes and doses, pathology, lesion scoring, viral antigen distribution in tissues

## Abstract

The understanding of the pathogenic mechanisms and the clinicopathological forms caused by currently circulating African swine fever virus (ASFV) isolates is incomplete. So far, most of the studies have been focused on isolates classified within genotypes I and II, the only genotypes that have circulated outside of Africa. However, less is known about the clinical presentations and lesions induced by isolates belonging to the other twenty-two genotypes. Therefore, the early clinicopathological identification of disease outbreaks caused by isolates belonging to, as yet, not well-characterised ASFV genotypes may be compromised, which might cause a delay in the implementation of control measures to halt the virus spread. To improve the pathological characterisation of disease caused by diverse isolates, we have refined the macroscopic and histopathological evaluation protocols to standardise the scoring of lesions. Domestic pigs were inoculated intranasally with different doses (high, medium and low) of ASFV isolate Ken05/Tk1 (genotype X). To complement previous studies, the distribution and severity of macroscopic and histopathological lesions, along with the amount and distribution of viral antigen in tissues, were characterised by applying the new scoring protocols. The intranasal inoculation of domestic pigs with high doses of the Ken05/Tk1 isolate induced acute forms of ASF in most of the animals. Inoculation with medium doses mainly induced acute forms of disease. A less severe but longer clinical course, typical of subacute forms, characterised by the presence of more widespread and severe haemorrhages and oedema, was observed in one pig inoculated with the medium dose. The severity of vascular lesions (haemorrhages and oedema) induced by high and medium doses was not associated with the amount of virus antigen detected in tissues, therefore these might be attributed to indirect mechanisms not evaluated in the present study. The absence of clinical signs, lesions and detectable levels of virus genome or antigen in blood from the animals inoculated with the lowest dose ruled out the existence of possible asymptomatic carriers or persistently infected pigs, at least for the 21 days period of the study. The results corroborate the moderate virulence of the Ken05/Tk1 isolate, as well as its capacity to induce both the acute and, occasionally, subacute forms of ASF when high and medium doses were administered intranasally.

## 1. Introduction

African swine fever (ASF) is a devastating viral haemorrhagic disease caused by a complex DNA arbovirus, the ASF virus (ASFV), the only member of the *Asfarviridae* family [1] that affects all members of the family Suidae [2]. ASF was first described in Kenya [3] where the virus spread from asymptomatic infected warthogs (*Phacochoerus aethiopicus*) to domestic pigs (*Sus scrofa*), causing high mortality rates in the pigs. Eurasian wild boar and feral pigs (*Sus scrofa*) succumb to ASFV and can transmit the virus to domestic pigs. No commercial vaccines against ASF are available. In 2007, ASF was present only in Africa and the island of Sardinia, Italy. However, the epidemiological situation changed dramatically with the incursion of ASFV genotype II into Georgia in 2007. From there, ASFV spread to the Trans Caucasus countries and the Russian Federation. In 2014, the virus entered in the European Union (EU), affecting the Baltic countries and continuing its expansion into Eastern Europe, with a long-distance introduction into Belgium in September 2018 [4]. In September 2020, Germany became the latest EU country to be affected [5]. Furthermore, in August 2018 the genotype II virus reached China [6], where almost half of the global pig population resides, spreading out of control to several countries in Asia and threatening the Australian continent [7]. Twenty-four distinct genotypes of ASFV have been described up to now [8,9], although only a few genome sequences have been published, of which only some have sufficient data available to enable in-depth genetic investigations [1,10]. In addition, studies to characterise the virulence and pathogenic mechanisms of isolates belonging to different genotypes have been mainly focused on viruses classified within genotype I [11,12,13,14,15,16,17,18,19,20] and genotype II [21,22,23,24,25,26], the only genotypes that have circulated outside of Africa, to date.

There are gaps in the basic knowledge of the biological and molecular features of the currently circulating ASFV isolates in Africa and Europe [27]. This knowledge is necessary to understand the pathogenic mechanisms and to characterise the clinicopathological forms caused by new ASFV isolates with potentially devastating consequences for the pork industry, exemplified by the case of the transcontinental spread of genotype II ASFV. Some studies have reported very low genetic variability in the circulating genotype II ASFV isolates in Europe and Asia since the incursion of the virus into Georgia in 2007 [10]. However, other studies have found genetic variations among genotype I ASFV isolates historically circulating in Western Africa, Europe and America, and also in genotype II ASFV isolates circulating in Europe and Eastern and Southern Africa [9,28,29,30]. It is unclear if the genetic variability of ASFV isolates classified within the different genotypes may influence the pathogenicity. For instance, previous experimental comparative infections in domestic pigs with the ASFV isolate Ken05/Tk1 (genotype X), used in the present study, and an isolate with a relatively similar genome sequence belonging to genotype IX (Ken06.Bus), revealed a marked difference in virulence [31].

Traditionally, ASFV isolates have been classified as highly virulent, responsible for peracute and acute forms of disease with case fatality up to 100%, moderately virulent, resulting in acute and subacute forms with reduced case fatality, and moderate to mildly virulent, associated with chronic or subclinical ASF [32]. Along with the virulence of the isolate, other factors such as age [20], dose and route of infection [19,33] have been pointed out as key for the evolution of clinical forms. Under natural conditions, ASFV is most transmitted among pigs via contact with excreted viral particles through nuzzling or ingestion, although tick bites, mechanical vectors, contaminated needles and other forms of parenteral infection are also involved [34]. Experimental oronasal inoculation mimics natural infection because it results in uptake of the ASFV via the oral and upper respiratory mucosa; the virus interacts with mucosal surfaces and is exposed to innate defence mechanisms [19]. Therefore, although oronasal infections may result in less reproducible clinical forms and more variability in clinical presentations than intramuscular inoculations, they constitute an optimal experimental inoculation route to study the early pathogenic mechanisms of disease and initial immune response against ASFV.

The present study provides detailed pathological data to complement previous studies that evaluated clinical and virological parameters in domestic pigs inoculated intranasally with different doses (high, medium and low) of the ASFV isolate Ken05/Tk1 [35]. This ASFV isolate was obtained from a tick extracted from a warthog burrow in central Kenya in 2005 [36]. The aims of this study were (i) to evaluate if the distribution and severity of macroscopic and histopathological lesions as well as the distribution of viral antigen in tissues were influenced by the different doses of virus administered and (ii) to propose and apply a comprehensive scoring system to evaluate macroscopic and histopathologic lesions, as well as the amount and distribution of viral antigen in tissue samples to facilitate standardized and harmonized comparison of ASF lesions across studies.

## 2. Results

**Figure 1:**
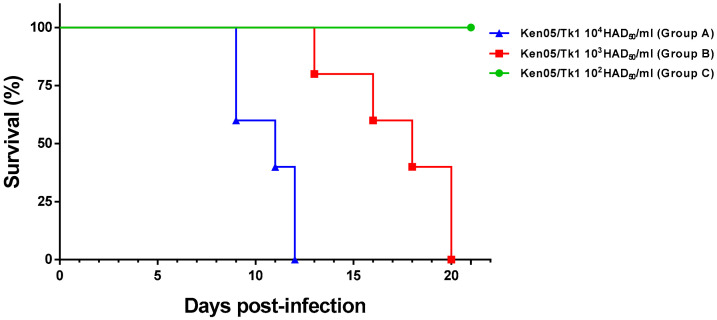
Evolution of mortality throughout the experiment. Fifteen pigs distributed in three groups (n = 5) we infected experimentally by intranasal route with 4.4 × 10^4^ HAD_50_/mL (group A—high dose), 4.4 × 10^3^ (group B—medium dose) and 4.4 × 10^2^ (group C—low dose) of ASFV isolate Ken05/Tk1 (genotype X). Days post infection (x-axis); percent survival (y-axis).

Pigs were euthanised upon reaching a predefined clinical score humane endpoint, at the end of the experiment (day 21 post inoculation) or to prevent housing of single animals (Figure 1).

### 2.1. Macroscopic Evaluation of Lesions

#### 2.1.1. Group A—High Dose (10^4^ HAD)

The intranasal inoculation of pigs in group A resulted in the direct infection of all pigs except for pig 88, which was euthanised at day 12 postinoculation (pi) due to ethical regulations to prevent housing of single animals. This animal showed late (from day 10 pi) and low viraemia levels, but not clinical signs before termination. The rest of the animals developed an acute form of ASF, displaying short incubation periods (ip), short clinical courses (cc) and viraemia from day 5 pi. Pigs 87 and 90 were the first that reached humane endpoint at day 9 pi after a short ip (5 days) and a short cc (5 days), followed by pig 86 at day 11 pi (ip: 7 days; cc: 5 days) and pig 89 at day 12 pi (ip: 6 days; cc: 7 days) (Table 1).

**Figure 2:**
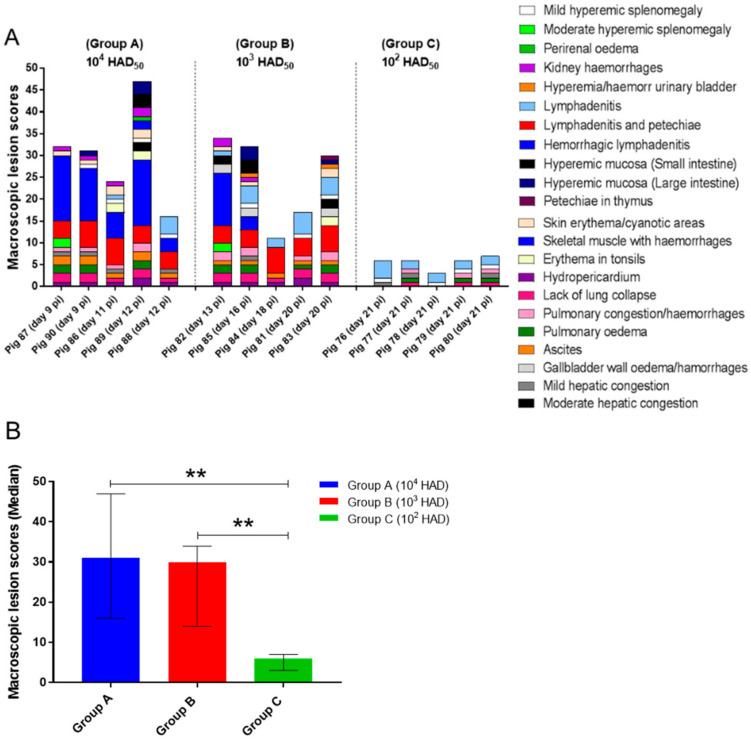
(**A**) Postmortem macroscopic lesion scoring. The tissues evaluated and the macroscopic lesions observed are displayed in the graph by different colours. Score of macroscopic lesions (y-axis); pigs evaluated in each experimental group (x-axis). (**B**) Median of cumulative macroscopic lesion scores (y-axis) in each experimental group (x-axis). Narrow bars indicate maximum and minimum values observed. Statistical analysis was carried out using the one-way ANOVA. Asterisks indicate statistically significant differences among experimental groups of pigs (** *p* < 0.01).

**Figure 3:**
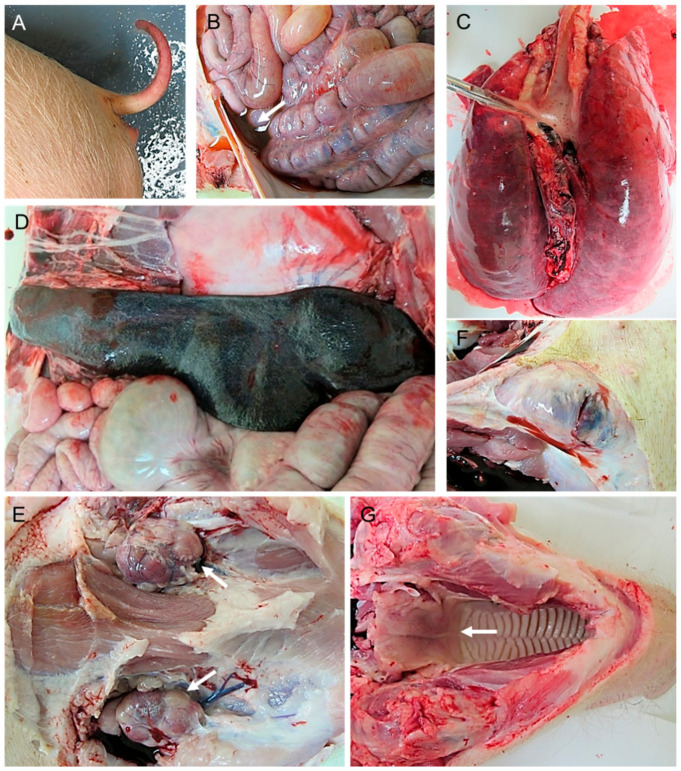
Macroscopic lesions observed in pigs infected by intranasal route with a high dose (4.4 × 10^4^ HAD_50_/mL) of ASFV isolate Ken05/Tk1 (genotype X). (**A**) Pig 86. Severe erythema and cyanosis along with petechial haemorrhages on the tail. (**B**) Pig 89. Moderate ascites with red-tinged fluid. (**C**) Pig 87. Severely congested, non-collapsed lungs with mild distension of interlobular septa (interstitial oedema) and severe alveolar oedema with the presence of foamy material in trachea. (**D**) Pig 87. Spleen with a moderate increase in size (moderate hyperaemic splenomegaly). (**E**) Pig 87. Lymphadenopathy and haemorrhages affecting submandibular lymph nodes. (**F**) Pig 89. Severe haemorrhagic lymphadenitis affecting superficial inguinal lymph nodes. (**G**) Pig 89. Moderate erythema in palatine tonsils.

While pig 88 only displayed mild lesions of ASF commonly described at initial stages (mild hydropericardium and ascites, mild hyperemic splenomegaly, lymphadenitis with petechial haemorrhages in renal lymph nodes and haemorrhagic lymphadenitis in mesenteric lymph nodes), the rest of the pigs showed more severe macroscopic lesions (Figure 2A). The most relevant lesions were the presence of cyanotic and haemorrhagic areas in the skin (tip of ears, chest, abdomen, tail, limbs and perianal area), which were more intense in pigs 86 (Figure 3A) and 89 euthanised at days 11 and 12 pi, mild (pigs 86, 87 and 90) to moderate (pig 89) hydropericardium, mild (pig 86) to moderate ascites (pigs 87, 89 and 90; Figure 3B) congested, non-collapsed lungs with interstitial and alveolar oedema along with the presence of abundant foam in trachea and bronchi (Figure 3C), mild congestion in the liver and petechial haemorrhages in the kidney (cortex and medulla). The spleens showed mild (pigs 86, 89 and 90) to moderate (pig 87; Figure 3D) hyperemic splenomegaly.

Haemorrhagic lymphadenitis was a common lesion, usually described in more than two lymph nodes per animal, that affected mainly retropharyngeal, submandibular (Figure 3E), tracheobronchial, mediastinal, gastrohepatic, renal and mesenteric lymph nodes. Other lymph nodes that also occasionally displayed this lesion were inguinal (Figure 3F) and cervical lymph nodes. Macroscopic scores were higher in pig 89 which, along with the lesions described, also showed erythema in tonsils (Figure 3G), haemorrhages in skeletal muscles, retroperitoneal oedema and hyperaemia of mucosal surfaces of small and large intestine.

#### 2.1.2. Group B—Medium Dose (10^3^ HAD)

**Figure 4:**
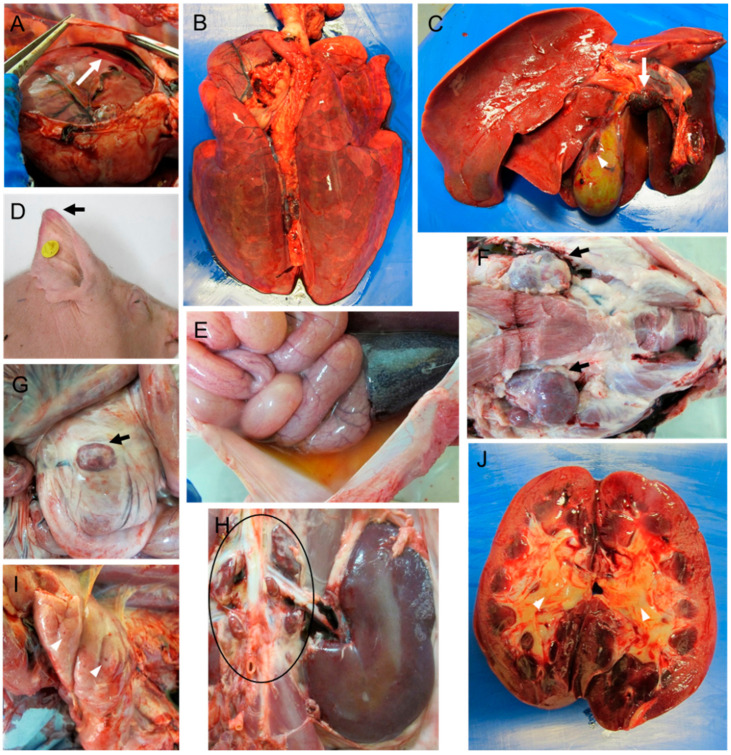
Macroscopic lesions observed in pigs infected by the intranasal route with a moderate dose (4.4 × 10^3^ HAD_50_/_mL_) of ASFV isolate Ken05/Tk1 (genotype X). (**A**) Pig 82. Moderate hydropericardium with reddish fluid. (**B**) Pig 82. Moderately congested, non-collapsed lungs with mild distension of interlobular septa (interstitial oedema). (**C**) Pig 82. Moderate hepatomegaly with diffuse congestion, haemorrhagic lymphadenitis of gastrohepatic lymph nodes (arrow) and haemorrhages (arrowhead) on the serosa surface of the gall bladder. (**D**) Pig 85. Erythema and cyanosis in the tips of ears. (**E**) Pig 83. Moderate ascites with yellowish fluid. (**F**–**H**) Pig 85. Lymphadenopathy and haemorrhages affecting submandibular lymph nodes (**F**), ileocaecal lymph node (**G**) and renal lymph nodes (**H**). (**I**) Pig 83. Multifocal petechial haemorrhages in thymus. (**J**) Pig 85. Kidney displaying mild congestion in renal cortex and medulla along with moderate oedema in renal pelvis (arrowheads).

Only 40% of the animals in group B (pigs 81 and 82) were infected after intranasal inoculation as indicated by viraemia levels and clinical signs. Both animals displayed a short incubation period (7 days). Pig 82 reached the humane endpoint at day 13 pi after a short clinical course (7 days) consistent with an acute form of ASF and displayed high levels of viraemia. On the other hand, pig 81 survived until day 20 pi after a long clinical course (14 days) consistent with a subacute form of ASF, displaying fluctuating moderate clinical signs and moderate viraemia levels (Table 1). It is worth noting that pig 81 did not reach the clinical endpoint scores and was euthanised to prevent single housing. Pig 82 displayed macroscopic lesions and scores similar to those described in most pigs from group A that developed a severe acute form of ASF (Figure 4A,B), also displaying a more congested liver, moderate to severe oedema of the gallbladder wall along with hyperaemia and haemorrhages in the gallbladder mucosa (Figure 4C). Meanwhile, although the clinical course of pig 81 was consistent with a subacute form of ASF, macroscopic scores (Figure 2A) were low, primarily characterised by the presence of generalized lymphadenitis with occasional presence of petechial haemorrhages or mild hyperemic splenomegaly, lesions characteristic of the moderate acute, rather than subacute, form of ASF.

As per their viraemia and clinical signs [35], pigs 83, 84 and 85 were indirectly infected by contact with infected animals after the intranasal inoculation of group B. Clinical signs were first observed at day 13 pi (pig 85), 15 pi (pig 84) and 17 pi (pig 83). Disease then developed rapidly and the animals were euthanised at day 16, 18 and 20 pi after a short clinical course of 4 days (Table 1). However, while pigs 83 and 85 displayed high viraemia levels at termination along with macroscopic lesions characteristic of severe acute forms of ASF (Figure 4D–J), pig 84 showed clinical alterations without temperature increase, very low viraemia levels and low macroscopic scores (Figure 2A), characterised by the presence of mild hydropericardium, mild ascites and generalized lymphadenitis with the presence of petechial haemorrhages. Pig 84 also displayed fibrinous pericarditis and fibrinous exudate on serosal surfaces of abdominal cavity organs, lesions that were associated with the presence of concomitant or secondary bacterial infections.

#### 2.1.3. Group C—Low Dose (10^2^ HAD)

All pigs in group C (10^2^ HAD) remained clinically normal throughout the experiment and the viral genome was not detected in blood samples of animals within this group. Pigs were euthanised at the end of the experiment (day 21 pi). Necropsies revealed the presence of nonspecific, mild macroscopic changes, such as lymphadenitis, affecting only some lymph nodes or hepatic congestion not suggestive or characteristic of ASF (Figure 2A).

Statistical analysis revealed significant differences among the macroscopic scores observed in groups A and B, regarding group C (*p* < 0.01), but not between groups A and B (Figure 2B).

### 2.2. Histopathological Evaluation of Lesions

Tissues samples from the three pigs with the highest macroscopic scores within each experimental group were selected for additional histopathological evaluations and immunohistochemical studies focused on determining viral antigen distribution. Tissue samples were collected from skin (ventrolateral abdominal area), lung (right cranial and caudal lobes), distal ileum, ileocaecal valve, colon, liver, kidney, spleen, thymus, tonsil, bone marrow and lymph nodes (submandibular, retropharyngeal, tracheobronchial, ileocaecal, gastrohepatic and renal).

#### 2.2.1. Group A—High Dose (10^4^ HAD) and Group B—Medium Dose (10^3^ HAD)

**Figure 5:**
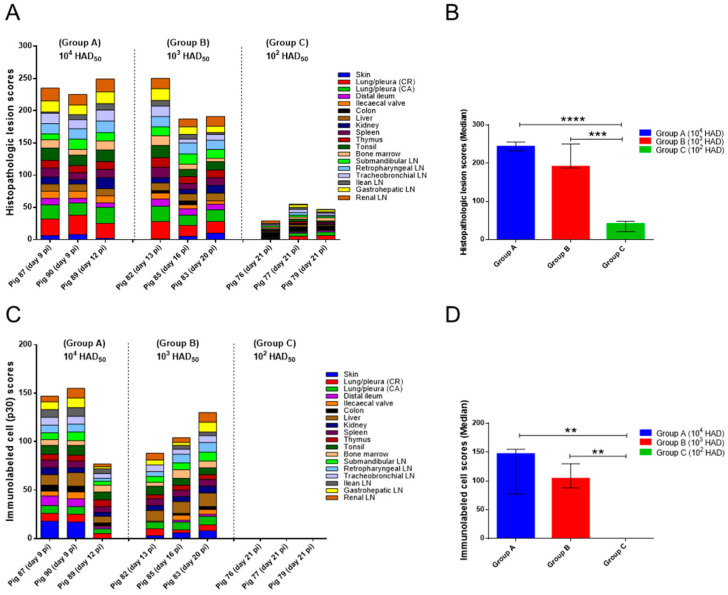
(**A**) Histopathologic lesion scoring. The tissues evaluated and the histopathologic lesions observed are displayed in the graph by different colours. Score of histopathologic lesions (y-axis); Pigs evaluated in each experimental group (x-axis). (**B**) Median of cumulative histopathologic lesion scores (y-axis) in each experimental group (x-axis). Narrow bars indicate maximum and minimum values observed. (**C**) Scores of cells immunolabeled for ASFV specific antigen p30/CP204L. The tissues evaluated and the scores of cells immunolabeled are displayed in the graph by different colours. Score of immunolabeled cells (y-axis); Pigs evaluated in each experimental group (x-axis). (**D**) Median of immunolabeled cell scores (y-axis) in each experimental group (x-axis). Narrow bars indicate maximum and minimum values observed. Statistical analysis was carried out using the one-way ANOVA. Asterisks indicate statistically significant differences among experimental groups of pigs (** *p* < 0.01; *** *p* < 0.001; **** *p* < 0.0001).

In concordance with macroscopic evaluations, the histopathological study revealed the severe lesions characteristic of ASF in all pigs evaluated in group A (87, 89 and 90) and B (82, 83 and 85). Histopathological scores (Figure 5A) were especially high in all pigs from group A infected with the highest dose of Ken05/Tk1 (10^4^ HAD) and in pig 82 from group B (10^3^ HAD).

**Figure 6:**
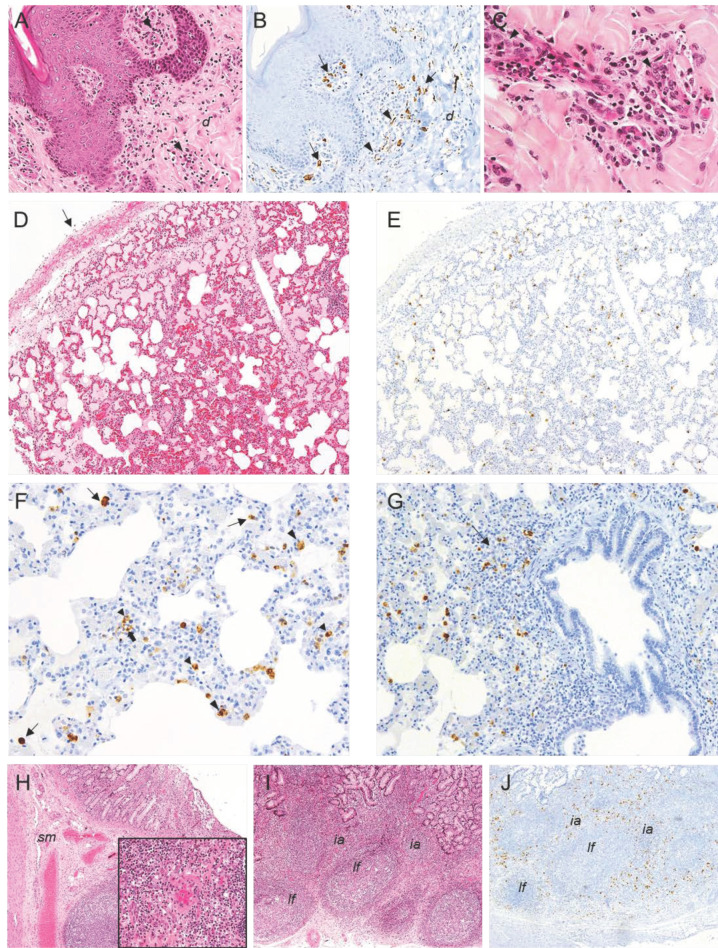
Histopathologic lesions observed in pigs infected by the intranasal route with high (4.4 × 10^4^ HAD_50_/mL) and moderate (4.4 × 10^3^ HAD_50_/mL) doses of ASFV isolate Ken05/Tk1 (genotype X). (**A**,**B**) Serial sections of skin; (**A**) HE stain, 20x. Mild perivascular mononuclear infiltrates (arrowheads) in dermis (*d*). (**B**) IHC, 20×. Macrophages (arrows) immunolabeled against viral antigen in perivascular infiltrates in dermis (*d*) along with endothelial cells positively immunolabeled (arrowheads). (**C**) HE stain, 40×. Capillary endothelial cells displaying hypertrophy with rounded nuclei (arrowheads) and vasculitis along with perivascular mononuclear infiltrates with the presence of pyknotic cells and cell debris. (**D**,**E**) Serial sections of lung. (**D**) HE stain, 10×. Moderate hyperaemia, septal thickening and alveolar proteinaceous oedema along with moderate pleural thickening (arrow). (**E**) IHC, 10×. Scattered cells immunolabeled against viral antigen in the pulmonary parenchyma. (**F**) IHC, 20×. Septal thickening and presence of alveolar macrophages (thin arrows), pulmonary intravascular macrophages (large arrowheads), interstitial macrophages (small arrowheads) and endothelial cells (thick arrow) immunolabeled for viral antigen. (**G**) IHC, 10×. Macrophages immunoreactive against viral antigen infiltrating the bronchus-associated lymphoid tissue (BALT) (arrow) and in the alveolar septa. (**H**) Distal ileum, HE, 4×. Mild diffuse hyperaemia in submucosa (*sm*); Insert: HE, 20×. Medium size vessel in submucosa with vasculitis. (**I**,**J**) Serial sections of distal ileum. (**I**) HE, 4×. Mild lymphocytic depletion of lymphoid follicles (*lf*). (**J**) IHC, 4×. Cells immunolabeled for viral antigen mainly in interfollicular areas (*ia*) and peripheral areas of lymphoid follicles (*lf*). Haematoxylin-eosin staining (HE); immunohistochemistry against protein (IHC); original magnification (number ×).

In the **skin** (Figure 6A,C), the lesions were mild and mainly observed in the dermis (hyperaemia with occasional minimal focal haemorrhages, vasculitis and minimal perivascular and intravascular accumulation of monocytes), although without significant differences between pigs in group A and B.

In the **lungs** (Figure 6D), along with the presence of diffuse moderate hyperaemia, angiectasia and thickening of alveolar septa, all pigs selected from group A and B also showed scattered mild alveolar haemorrhages, diffuse moderate alveolar oedema along with the presence of cell debris, fibrin deposits, macrophage infiltrates and the occasional presence of megakaryocytes in alveolar lumen. Some small and medium vessels showed mild oedema in tunica media along with prominent diffuse endothelial activation characterised by endothelial cell hypertrophy and rounded nuclei. Moderate, scattered peribronchial and peribronchiolar mononuclear infiltrates were also described. Pig 90 also showed microthrombosis in some vessels. Pigs infected with the highest dose (Group A) displayed diffuse, moderate to severe interstitial oedema, along with mild pleural thickening and occasional presence of pleural or subpleural haemorrhages, histopathological changes that were more severe than those observed in pigs from group B.

**Figure 7:**
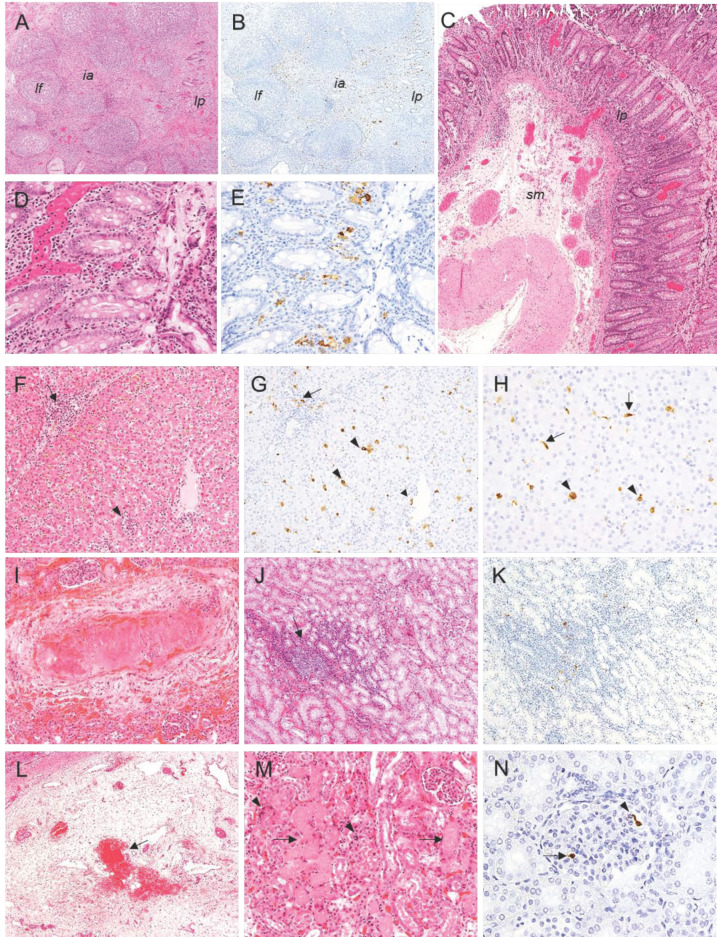
Histopathologic lesions observed in pigs infected by the intranasal route with high (4.4 × 10^4^ HAD_50_/mL) and moderate (4.4 × 10^3^ HAD_50_/mL) doses of ASFV isolate Ken05/Tk1 (genotype X). (**A**,**B**) Serial sections of ileocaecal valve. HE, IHC, 4×. Mild diffuse hyperaemia in submucosa, mild lymphocytic depletion of lymphoid follicles and cells immunolabeled for viral antigen mainly in lamina propria (*lp*), interfollicular areas (*ia*) and peripheral areas of lymphoid follicles (*lf*). (**C**) Colon HE, 4×. Moderate mononuclear infiltrates in lamina propria (*lp*) and moderate hyperaemia and oedema in the submucosa (*sm*). (**D**,**E**) Serial sections of colon. HE, IHC, 20×. Mononuclear infiltrates in lamina propria along with the presence of cells, mainly macrophages, immunolabeled against viral antigen. (**F**,**G**) Serial sections of liver. HE, IHC, 10×. Interstitial mononuclear infiltrates in portal spaces (arrow) and hepatic parenchyma (small arrowhead) along with sinusoidal leukocytosis. Observe the presence of mononuclear cells in the infiltrates (arrow), hepatocytes (large arrowheads) and endothelial cells (small arrowheads) immunolabeled against viral antigen. (**H**) Liver. IHC, 20×. Presence of enlarged Kupffer cells (arrows) and hepatocytes (arrowheads) immunolabelled positively for viral antigen. (**I**) Kidney. HE, 10×. Severe diffuse interstitial congestion, multifocal haemorrhages along with necrotizing vasculitis with presence of microthrombi and perivascular oedema in renal cortex. (**J**,**K**) Serial sections of kidney. HE, IHC, 10×. Interstitial inflammatory mononuclear infiltrates (arrow) in renal cortex and cells positive for viral antigen, mainly macrophages, within the inflammatory infiltrates. (**L**) Kidney. HE, 4×. Severe focal interstitial haemorrhage (arrow) and diffuse moderate interstitial oedema in renal pelvis. (**M**) Kidney. HE, 10×. Tubulonephrosis (arrows) and focal tubular necrosis (arrowheads) in renal cortex. (**N**) Kidney. IHC, 20×. Presence of a circulating monocyte (arrow) and a capillary endothelial cell (arrowhead) immunolabeled against viral antigen within a glomerulus. Haematoxylin-eosin staining (HE); immunohistochemistry against p30/CP204L protein (IHC); original magnification (number ×).

The differences in the severity of the lesions described in the **distal ileum** (mild diffuse hyperaemia in submucosa with presence of small and medium size vessels with intramural inflammatory infiltrates (Figure 6H), mild diffuse lymphocytic depletion of Peyer’s patches and interfollicular areas (Figure 6I) were not remarkable between the pigs from groups A and B. However, vascular changes, lymphocytic depletion and cellular fragmentation phenomena observed in the **ileocaecal valve** sections (Figure 7A) were more severe in pigs infected with the highest dose of Ken05/Tk1 (group A). In the **colon**, lesions in pigs in group A were also more severe and characterised by the presence of mild diffuse hyperaemia with occasional haemorrhages in the epithelium, the presence of mild multifocal mononuclear infiltrates in lamina propria (Figure 7C,D) and severe hyperaemia and oedema in submucosa.

In the **liver**, similar lesions were observed in pigs from group A and B. Histopathological changes (Figure 7F) were characterised by the presence of mild, diffuse congestion and expanded hepatic sinusoids, moderate, diffuse interstitial lymphoplasmacytic infiltrates in portal spaces and interlobular areas with presence of mild cellular fragmentation, scattered, small foci of lymphoplasmacytic infiltrates in hepatic sinusoids, moderate, diffuse sinusoidal leukocytosis with presence of enlarged Kupffer cells in hepatic sinusoids and small, scattered, necrotic foci of hepatocytes. Prominent endothelial activation was accompanied by vasculitis with intramural inflammatory mononuclear infiltrates and mononuclear perivasculitis that affected mainly arteries and veins in portal spaces.

Pigs from group A showed more severe lesions in the **kidney** than pigs in group B, characterised by the presence of mild to moderate, diffuse congestion and multifocal, interstitial haemorrhages in both the renal cortex (Figure 7I), medulla and renal pelvis (Figure 7L). Pig 87 (group A) also had evident interstitial oedema in the renal cortex. Multifocal interstitial mononuclear infiltrates, mainly observed in the renal cortex (Figure 7J), were also more severe in pigs from group A, which also displayed protein rich fluid in Bowman’s space and tubules due to the leakage from damaged glomeruli. Pigs 83 (group B) and 87 (group A) also displayed focal, fibrinonecrotic glomerulonephritis, focal, mild to severe tubulonephrosis with intratubular hyaline droplets as well as focal tubular necrosis (Figure 7M). Microthrombi, along with intramural inflammatory infiltrates, were also observed in these pigs, along with severe, focal, necrotizing vasculitis of renal arterioles in pig 89 (Figure 7I).

**Figure 8:**
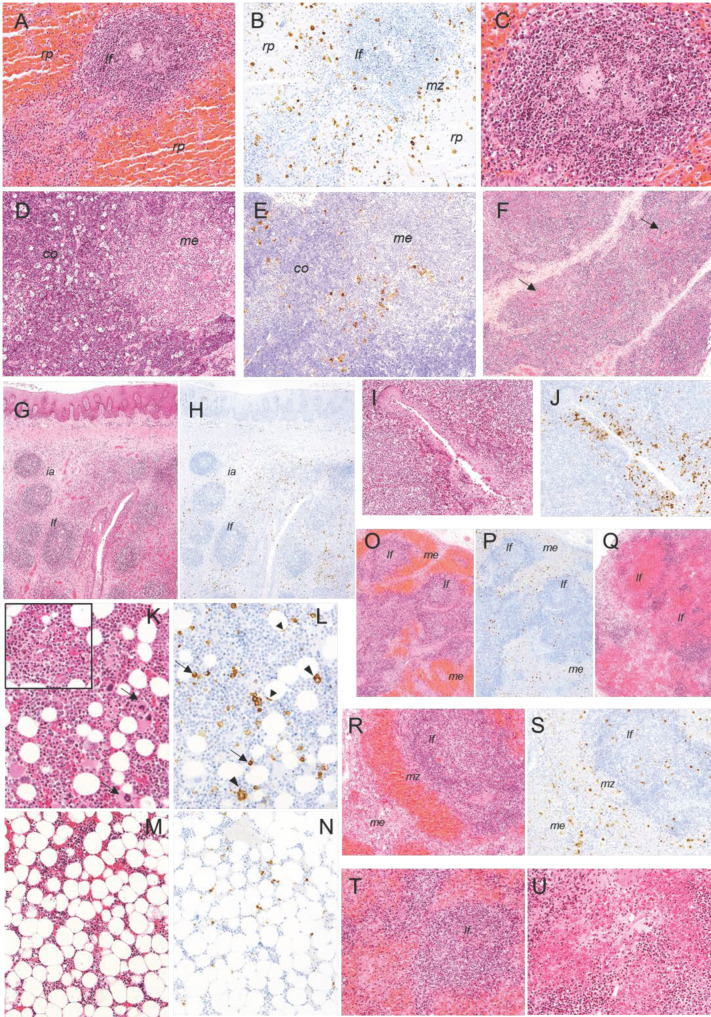
Histopathologic lesions observed in pigs infected by the intranasal route with high (4.4 × 10^4^ HAD_50_/mL) and moderate (4.4 × 10^3^ HAD_50_/mL) doses of ASFV isolate Ken05/Tk1 (genotype X). (**A**,**B**) Serial section of spleen. HE, IHC, 10×. Severe, diffuse engorgement of the splenic red pulp (*rp*), lymphoid follicle (*lf*) infiltrated by erythrocytes displaying moderate lymphoid depletion and presence of a high number of cells, mainly hypertrophic macrophages, immunolabelled against viral antigen in the splenic red pulp (*rp*), marginal zone (*mz*) and within lymphoid follicles (*lf*). (**C**) Spleen. HE, 20×. Detail of lymphoid follicle showing moderate lymphoid depletion along with abundant pyknotic cells, cell debris and macrophages infiltrates. (**D**,**E**). Serial sections of thymus. HE, IHC, 10×. Severe, diffuse presence of tingible body macrophages with abundant cytoplasm containing cell debris in cortex (*co*), giving rise to the so-called ‘starry sky’ appearance. Cells immunolabeled for viral antigen, mainly macrophages, in cortex (*co*) along with the presence of macrophages and star-shaped cells in the medulla (*me*) and corticomedullary junction. (**F**) Thymus, HE, 10×. Severe atrophy of cortex and presence of Hassall’s corpuscles (arrows) at the surface of the organ. (**G**,**H**) Serial sections of palatine tonsils. HE, IHC, 4×. Moderate lymphoid depletion in lymphoid follicles (*lf*) and interfollicular areas (*ia*). Cells, mainly macrophages and some lymphocytes, immunolabeled for viral antigen in interfollicular areas and around lymphoid follicles. (**I**,**J**) Serial sections of palatine tonsil. HE, IHC, 10×. Detail of a crypt showing a severe infiltrate of mononuclear cells, which display pyknosis and cell fragmentation. Observe the massive infiltration of cells, mainly macrophages and lymphocytes, immunolabeled for viral antigen in the epithelium of the crypt. Serial sections of bone marrow. HE, IHC, 20× (**K**,**L**) and 10× (**M**,**N**). Observe the severe hypocellularity and the scarce number of megakaryocytes (arrows) in (**M**) compared with (**K**). Insert (HE, 20×). Presence of pyknotic cells, cell fragmentation and fibrin strands. Viral antigen was mainly detected in monocytes and macrophages (arrows) and, occasionally in megakaryocytes (large arrowheads) and capillary endothelial cells (small arrowheads). (**O**,**P**) Serial sections of renal lymph node. HE, IHC, 4×. Moderate lymphoid depletion of lymphoid follicles (*lf*) and diffuse lymphoid tissue along with severe haemorrhages in medullary cords. Cells immunolabeled for viral antigen were mainly observed in the medulla (*me*) and interfollicular areas of the cortex. The presence of cells immunolabelled within lymphoid structures was lower. (**Q**) Gastrohepatic lymph node. HE, 4×. Severe lymphoid depletion of lymphoid follicles, which appear massively infiltrated by erythrocytes. (**R**,**S**) Serial sections of renal lymph node. HE, IHC, 10×. Detail of lymphoid follicle (*lf*) displaying moderate lymphoid depletion along with the presence of abundant pyknotic cells, cell fragmentation and macrophage infiltrates. Observe the high number of cells immunolabeled against viral antigen mainly in medulla (*me*) and marginal zone (*mz*) of the lymphoid follicle. (**T**) Gastrohepatic lymph node. HE, 10×. Lymphoid follicle (*lf*) displaying severe lymphoid depletion and infiltration of erythrocytes. (**U**) Renal lymph node, HE, 20×. Vasculitis and severe perivascular haemorrhages. Haematoxylin-eosin staining (HE); immunohistochemistry against p30/CP204L protein (IHC); original magnification (number ×).

In the **spleen**, all pigs in group A (87, 89, 90) and pig 82 in group B showed severe, diffuse lymphocytic depletion of splenic white pulp along with a severe, diffuse, engorgement of red pulp (Figure 8A). The splenic red pulp appeared full of mononuclear cells, cell debris and fibrin deposits. The presence of megakaryocytes was also observed. Lymphoid follicles and periarteriolar lymphoid sheaths were small and difficult to discern, appearing to be infiltrated by erythrocytes and macrophages, many of them with abundant cytoplasm containing phagocytized cell debris (tingible bodies), and showing a severe, lymphocytic depletion along with abundant pyknotic cells and cell debris (Figure 8C).

In the **thymus**, cortex atrophy was the most significant change observed. Most of the pigs in group A and B showed mild to moderate, diffuse lymphocytic depletion. The cortex appeared full of pyknotic cells and cell fragmentation along with a moderate to severe, diffuse presence of tingible body macrophages (TBM) (Figure 8D). Occasional small haemorrhages were also present. In pig 89 (group A), lymphocytic depletion was especially severe, resulting in the prominence of Hassall’s corpuscles at the surface of the organ (Figure 8F), while images of cell debris and TBM were less frequent.

The **tonsils** showed diffuse, moderate to severe lymphocytic depletion (Figure 8G) that affected both lymphoid follicles and interfollicular areas, changes that were more severe in pigs of group A (87, 89 and 90) and in pig 82 (group B). Lymphocytic depletion was accompanied by the presence of pyknotic cells, cell fragmentation and TBM infiltrates, the latter especially abundant within the lymphoid follicles. Connective tissue proliferation was also a characteristic change observed in interfollicular areas. The epithelium of the crypts showed moderate to severe infiltrates of mononuclear cells which also displayed pyknosis and cell fragmentation (Figure 8I).

In the **bone marrow** diffuse, moderate to severe hypocellularity with occasional presence of fibrin strands, focal haemorrhages (Figure 8K,M), endothelial activation in small and medium size vessels and a low number of megakaryocytes were observed. The changes were especially marked in pig 82 (group B) and 89 (group A). Pyknosis and cell fragmentation were scarce.

Finally, all pigs in groups A and B showed severe histopathological lesions in the **submandibular, retropharyngeal, gastrohepatic and renal lymph nodes** (LNs). All pigs in group A (87, 89, 90) and pig 82 in group B also showed severe histopathological lesions in the **tracheobronchial lymph nodes,** while lesions in the **ileocaecal lymph nodes** were mild to moderate. Diffuse, moderate to severe haemorrhages that mainly affected medullary cords and diffuse lymphoid tissue in the cortex were observed. Marginal zones of lymphoid follicles in the cortex and germinal centres usually were infiltrated with blood. In addition, there was moderate to severe lymphocytic depletion that affected both the lymphoid follicles and diffuse lymphoid tissue, accompanied by a massive presence of pyknotic cells and cell fragmentation, which was also observed in the medulla (Figure 8O,Q,R,T). Megakaryocytes were usually observed among haemorrhages and cell debris. Capillary and small-vessel endothelial cells displayed cellular hypertrophy and the rounded, prominent nuclei characteristic of endothelial activation. Occasionally, vasculitis with intramural inflammatory infiltrates, necrosis of endothelial cells, perivascular oedema and fibrin deposits in the vascular lumina were also observed (Figure 8U).

#### 2.2.2. Group C—Low Dose (10^2^ HAD)

In group C (10^2^ HAD), the histopathological evaluation revealed nonspecific lesions and mainly mild vascular changes in the **lung**, **liver** and **kidney** of pigs 77 and 79 (Figure 5A). Pig 77 also showed mild histopathological changes in the **thymus** (cortical atrophy and lymphocytic depletion in the medulla) as well as in the **tonsils** and some **lymph nodes** (retropharyngeal, tracheobronchial, ileocaecal and gastrohepatic), which displayed mild, diffuse hyperaemia with occasional haemorrhages along with mild to moderate lymphocytic depletion that affected mainly lymphoid follicles.

Statistical analysis revealed significant differences in the histopathological scores of groups A and B with respect to group C, but not between groups A and B (Figure 5B).

### 2.3. Evaluation of Viral Antigen Distribution

The presence of cells immunolabeled for the ASF-specific antigen p30/CP204L was not detected in any of the tissues taken from the pigs included in group C (10^2^ HAD). On the other hand, the viral antigen was detected in most of the tissues evaluated from pigs in groups A and B. Pigs 87 and 90 (group A) and pig 83 (group B) displayed the highest presence of immunolabeled cells (Figure 5C). However, statistical analysis did not reveal significant differences regarding immunolabeled cells between groups A and B (Figure 5D). Circulating monocytes and macrophage populations of different tissues, along with endothelial cells of small vessels and capillaries, were the main target cells of the virus, with high numbers of cells immunolabeled in lungs, liver and lymphoid organs.

Immunolabeled cells, mainly endothelial cells, circulating monocytes, macrophages within perivascular infiltrates and reticular cells, were observed in the dermis of **skin** sections (Figure 6B), with pigs 87 and 90 (group A) displaying particularly high numbers.

In the **lungs**, viral antigen was detected mainly in the pulmonary intravascular macrophages (PIMs) and, to a lesser degree, in the pulmonary alveolar macrophages (PAMs). Endothelial cells of small vessels and capillaries were also immunolabeled (Figure 6E,F). The viral antigen was also detected in pneumocytes. Positive cells, mainly macrophage-like cells, were occasionally observed infiltrating the bronchus-associated lymphoid tissue (BALT) of some animals (Figure 6G). Differences regarding the nature and amount of immunolabeled cells were not observed between cranial and caudal lobes.

A high number of positive cells was observed in some intestinal areas of pigs 87 and 90 (group A). Immunolabeled cells, mainly macrophages and occasionally lymphocytes, were located in the lamina propria and interfollicular areas of the submucosa of the **distal ileum** (Figure 6J) and **ileocaecal valve** (Figure 7B), and also in the lamina propria and submucosa of the **colon** (Figure 7E).

In the **liver**, circulating monocytes and Kupffer’s cells within hepatic sinusoids, macrophages within interstitial infiltrates, mainly in portal spaces and interlobular areas, along with a high number of hepatocytes, were immunolabeled. Endothelial cells immunolabeled for viral antigen were also observed mainly in capillaries and small-size vessels in portal spaces, hepatic sinusoids and, occasionally, in central veins (Figure 7G,H).

In the **kidney**, antigen-positive cells, mainly macrophages, were observed within the multifocal interstitial mononuclear infiltrates of the renal cortex and medulla (Figure 7K). Within the glomeruli, capillary endothelial cells, circulating monocytes and mesangial cells were also occasionally immunolabeled (Figure 7N). The presence of immunolabeled cells was especially high in pig 87 (group A) and pigs 83 and 85 (group B).

In the **spleen**, red pulp showed a massive presence of immunolabeled macrophages, most of them showing an increase in size. Lymphocytes labeled for viral antigen were also frequently observed in this area. Immunolabeled cells also appeared in peripheral areas of splenic lymphoid follicles and less frequently within lymphoid follicles (Figure 8B). Occasionally, star-shaped cells, likely reticular or follicular dendritic cells, were positive for viral antigen within splenic lymphoid follicles.

In the **thymus**, macrophages, which also showed an increase in size, were the main target cells. The presence of positive cells was especially high in the corticomedullary junction. Occasionally, lymphocytes and star-shaped cells in both the cortex and medulla were also immunolabeled (Figure 8E).

In the **tonsils**, the highest number of cells immunolabeled for viral antigen, mainly macrophages and some lymphocytes, was observed infiltrating the epithelium of the crypts and in the diffuse lymphoid tissue around the crypts. Tonsillar epithelium was also positive. A high number of labeled cells, mainly macrophages and some lymphocytes, were also observed in interfollicular areas, while the presence of antigen positive cells inside lymphoid follicles was lower (Figure 8H,J). Immunoreactive star-shaped, likely reticular or dendritic cells, were also observed in interfollicular areas and within lymphoid follicles.

Myeloid cells were the main target cells in the **bone marrow**, while the presence of immunolabeled megakaryocytes and granulocytic cells was low (Figure 8L,N).

Regarding the **lymph nodes**, the ileocaecal lymph nodes generally showed a lower presence of immunoreactive cells. The rest of the evaluated lymph nodes displayed a high number of immunolabeled cells, mainly macrophages and some lymphocytes, that were mainly observed in the medulla and interfollicular areas of the cortex, while the presence of positive cells within the lymphoid follicles was low (Figure 8P,S). Star-shaped, immunolabeled cells were also observed occasionally in interfollicular areas and within lymphoid follicles. In areas with severe haemorrhages and lymphocytic depletion, the presence of immunolabeled cells was very low.

## 3. Discussion

As reported previously [35], the intranasal inoculation of the Ken05/Tk1 isolate with a dose of 4.4 × 10^4^ HAD resulted in the direct infection of 80% of the animals (four out of five pigs). The pigs developed typical acute forms of ASF [32,37,38] characterised by short clinical courses (from 5 and 7 days) before reaching the clinical endpoint between day 9 and 12 pi. Even pig 88, likely infected indirectly by contact with pen mates and euthanised at day 12 pi due to ethical considerations but without clinical signs, showed viraemia levels and macroscopic lesions commonly described in the initial stages of acute ASF forms [32,33]. These changes may have agreed with an outcome equivalent to that of its pen mates within the next 2 to 4 days.

In contrast, only 40% of the animals (two out of five pigs) were directly infected after the administration of a 10-fold lower dose (4.4 × 10^3^ HAD). These animals also showed slightly longer incubation periods of 7 days and lower and delayed viraemia levels than those detected in most of the pigs directly infected after the administration of a higher dose. On the other hand, the clinical and virological data observed from day 12–13 pi suggested that the other three of the five animals within this group inoculated with 10^3^ HAD did not actually develop a long incubation period, but rather became infected indirectly by contact with their pen mates [35]. Regardless of whether pigs inoculated with 10^3^ HAD had become infected directly or indirectly, all except one (pig 81) developed a short clinical course (from 4 to 7 days) before euthanasia as well as macroscopic lesions similar to those described in pigs from group A infected with 10^4^ HAD and characteristic of acute forms of ASF [32,37,38].

As mentioned, only pig 81 developed a long clinical course (14 days) with fluctuating clinical signs and moderate viraemia levels. Such a clinical course was consistent with a subacute, rather than acute, form of ASF. However, this animal was euthanised before reaching a humane endpoint due to ethical considerations to prevent single animal housing. This pig showed lesions and lower macroscopic scores that corresponded to acute, rather than subacute, forms of ASF [32]. If this pig had been allowed to live longer, two possible outcomes would have been plausible. Pig 81 might have recovered or, alternatively, a moderate clinical course with fluctuating clinical signs and moderate viraemia levels could have been extended until an ultimately fatal outcome where lesions may then have been more consistent with a subacute form. Animals that develop this kind of subacute course of disease usually display more severe and widespread haemorrhages and oedema, and therefore higher gross pathology scores, than those described in acute forms [32,38]. Subacute lesions are usually accompanied by severe leukopenia, severe thrombocytopenia and erythrodiapedesis due to vasodilation phenomena [13,39,40]. Finally, it is important to note that the presence of high lesion scores in pigs may not be directly linked to highly virulent isolates because acute ASF forms induced by highly virulent isolates usually display fewer lesions than subacute forms induced by moderately virulent isolates, where widespread haemorrhages and oedemas are frequent.

Preliminary, but independent, studies where domestic pigs, 10–12 weeks old, were inoculated by intramuscular [31] and intranasal route [41] with a low dose (10 HAD) were carried out to determine the virulence of the Ken05/Tk1 isolate. The results suggested that the route of infection influenced the evolution and outcome of the clinical forms. In such studies, animals inoculated intranasally that did not reach the end of the experiment (euthanised or died between day 12 and 21 pi) showed a delay in both the appearance of viraemia and clinical signs (between day 7 and 14 pi). This was coupled with longer clinical courses than pigs infected intramuscularly which became viremic from day 3 pi, displayed clinical signs from day 6–7 pi and were euthanised or died between day 11 and 18 pi. In the previous experiments, regardless the route of infection, 50% of animals survived until the end of the experiments, displaying both mild clinical signs and low viraemia levels. Although pigs infected intramuscularly that reached the end of the experiment (day 70 pi) displayed the severe haemorrhagic lesions characteristic of subacute ASF, along with a high presence of infected cells detected by immunohistochemistry in tissues [31], the pigs intranasally inoculated showed no lesions and hardly any presence of infected cells in tissues [41]. These results suggest a possible role of mucosa -associated lymphoid tissue (MALT) in the selective absorption of the virus, as well as in the induction of an immune response capable of modulating and controlling virus replication and spread [33,42]. ASFV replication in MALT has been detected as early as 24 h pi and prior to systemic dissemination [19,43,44]. However, the nature of local immune responses triggered in MALT at initial virus replication stages and their systemic consequences are unclear. Further comparative studies, between highly and moderately virulent isolates, focusing on the initial stages of infection are required to understand these mechanisms.

In previous studies, after intranasal infections with a low dose of Ken05/Tk1 (10 HAD), 50% of pigs showed viraemia, clinical signs and were euthanised displaying characteristic lesions of subacute ASF [41]. In contrast, in the present study all animals inoculated intranasally with the lowest dose (10^2^ HAD) survived until the end of the experiment without showing clinical signs, lesions or detectable levels of the virus genome in blood. Such differences may be attributed not only to experimental design and the titration methods used by different laboratories, but also to other factors likely associated with the nature and pathogenicity of moderately virulent isolates that can lead to a greater level of variation in results. Similar variable results have been described in the course of experimental infections with other moderately virulent ASFV isolates that were administered intranasally at the same dose (3.5 log_10_ median tissue culture infective dose-TCID_50_/mL) to domestic pigs of the same age (12 weeks old). In these experiments, mortality rates ranged from 0% [18] to 100% by day 15 pi [20].

The difficulty of carrying out comparative intranasal inoculations with medium and high doses of moderately virulent ASFV isolates has been previously highlighted [18]. In that study, three groups of domestic pigs (12 weeks old) were housed in separate compartments and five pigs in each group were inoculated intranasally with a moderately virulent isolate, either the Malta’78 (3 log_10_ TCID_50_) (medium dose) or 4 log_10_ TCID_50_ (high dose) per pig) or the Netherlands’86 (3.5 log_10_ TCID_50_ per pig) isolate. In both the Malta’78 medium dose group and the Netherlands’86 group, the inoculation was successful in three of the five pigs, and the remaining pigs were considered contact exposed. In the Malta’78 high dose group, the inoculation was successful in all five pigs. Inoculation successes were similar to those described with medium and high doses of Ken05/Tk1 in the present experiment. However, while most of pigs inoculated with Malta’78 and Netherlands’86 survived up to day 70 pi, showing low viraemia levels but not clinical signs, pigs inoculated with medium and high doses Ken05/Tk1 developed clinical courses and had to be euthanised between day 9 and 20 pi, suggesting a higher virulence than for the Malta and Netherlands isolates.

Most of the information available regarding isolates classified within genotype X is focused on their molecular characterization [36,45,46], with few references to experimental studies focused on evaluating their virulence and clinical courses in pigs. In a comparative experimental infection, indigenous domestic African pigs and European genetic lineage pigs of the same age (6 months old) were inoculated intranasally with 10 HAD of ASFV Ken05/K2 domestic pig isolate [47]. Indigenous African pigs displayed a delay in the incubation period and in the appearance of viraemia. In contrast to European lineage pigs, which displayed high temperatures and died or were euthanised between day 13 and 21 pi, indigenous African pigs showed inconsistent clinical signs without high temperatures and died or were euthanised between day 18 and 32 pi. During macroscopic evaluations, indigenous African pigs displayed more severe and widespread haemorrhages and oedemas than European lineage pigs, which were characteristic of subacute forms of ASF. Virus titers in the spleen and lymph nodes were also lower than those detected in European lineage pigs. These results demonstrated that in addition to other factors like age, route of infection, dose and virulence of the ASFV isolates [20,32], breed and the genetic background of the host also play a key role in the evolution of ASF clinical forms.

Unusual or unexpected virological and immunological responses against genotype X isolates have also been discussed in different studies. Three of ten European domestic pigs oro-nasally infected with the moderately virulent ASFV isolate Ken05/K2 did not exhibit an antibody response whilst in the remaining animals, an antibody response was initiated between days 10 and 23 pi. [48]. Other studies carried out in East Africa described the presence of apparently asymptomatic local domestic pigs positive for ASFV by PCR in tissues (virus isolates belonging to genotypes IX and X), but negative for ASFV in blood by PCR and serology. These results suggest a potential role of carrier domestic pigs as a source of infection [49,50]. However, whether animals positive for ASFV by PCR in tissues were also capable of transmitting the disease to uninfected, naïve pigs was not investigated. It has been suggested that ASFV may persist for long periods in tissues or blood from recovered pigs or following infection with moderate-to-low virulence isolates, contributing to disease persistence in endemic areas and causing sporadic outbreaks or introduction into disease-free zones [51,52].

On the other hand, an extensive literature review found no evidence of a role for long-term carriers in transmission [53]. In the present experiment, in addition to the absence of detectable levels of the virus genome in blood samples from pigs infected with the lowest dose (10^2^ HAD) of Ken05/Tk1 throughout the experiment, the absence of viral antigen in tissue samples did not support the existence of possible carriers or persistently infected pigs, at least for the 21 day period of the study.

The evaluation protocols used in the present study were modified and adapted from previous protocols [37] to include (i) a simplified pathological evaluation criteria, accompanied by a gross lesions scoring chart that was easy to fill out; (ii) a broader scoring system including a wider number of organs; (iii) a precise scoring system that includes scoring of lesions typically described in the subacute and chronic forms of ASF, not evaluated in previous protocols; (iv) the addition of a protocol to score results of IHC. The protocols proposed allowed accurate standardized pathological assessments to be carried out and contributed to faster evaluation procedures at necropsy. The scoring sheets provided in the Supplementary Materials are simple and self-explanatory. Additionally, the integration of all scorings (clinical, macroscopic lesions, histopathological lesions and immunohistochemical ASFV specific staining) provided a thorough picture of the presentation and severity of disease in individual animals. Semiquantitative scorings also allowed comparison between animals and groups.

Regarding the pathological evaluations, two pigs inoculated with medium and high doses of Ken05/Tk1 (pigs 82 and 89) that displayed severe lesions and the highest macroscopic and histopathological scores, showed the lowest number of cells immunolabeled for viral antigen in tissues. This fact corroborates that the severity and extension of lesions may not be directly related with the amount of virus present in the tissue but with indirect mechanisms triggered by ASFV as previously suggested [38,54]. Because lesions and viral antigen distribution in tissues were only evaluated in samples taken when the humane endpoint was reached and not at early stages, viral spread and the evolution of the pathogenic mechanisms of lesions induced by the Ken05/Tk1 isolate require further study. The viral antigen was detected in all the tissues evaluated from the pigs selected in the groups infected with the medium and high doses without significant differences in the amount of immunolabeled cells. Along with the monocytes−macrophages, which are considered to be the main ASFV target cells [12,17,55], and the endothelial cells of small vessels and capillaries, many other cells were immunolabeled for viral antigen p30/CP204L and morphologically identified in the selected tissues. Among them were hepatocytes, lymphocytes, star-shaped cells (likely reticular or dendritic cells), tonsillar epithelium, megakaryocytes and granulocytic cells. Infection or replication of ASFV in these cells usually takes place only in the final stage of the disease without any apparent major role in the pathogenesis of the disease. Contradictory studies have suggested that the expression of specific markers associated with the maturation stages of macrophages, such as CD163, might increase their susceptibility to ASFV infection [56,57]. It has also been speculated that infection of nonmacrophage cells might be favoured by the expression of ASF virus-membrane receptors when monocytes−macrophages are still present and undergoing intense proliferation [38,58]. However, both the mechanisms and the role of receptors regarding a cell’s susceptibility to ASFV infection remain unclear.

## 4. Materials and Methods

### 4.1. Experimental Design, Clinical Evaluations, Sampling and Quantification of Viral DNA

Details of experimental design, clinical evaluations, sampling and quantification of viral DNA levels in blood samples from the domestic pigs presented in this study have been previously described [35]. Animal experiments were carried out in the biosafety level 3 (BSL3) facilities at Animal and Plant Health Agency (APHA, Weybridge, UK). Fifteen Large White pigs were housed and grouped into three groups of five animals. Animals were inoculated at 12 weeks of age, after a period of acclimatisation, using intranasal mucosal atomisation devices (MAD Intranasal, Teleflex), with 2 mL (1 mL per nostril) of ASFV isolate Ken05/Tk1. The doses inoculated were 4.4 × 10^4^ HAD_50_/mL (group A—high dose; pigs 86 to 90), 4.4 × 10^3^ (group B—medium dose; pigs 81 to 85) and 4.4 × 10^2^ (group C—low dose; pigs 76 to 80), respectively. To simplify, throughout the manuscript we will refer to the experimental groups as group A (10^4^ HAD), group B (10^3^ HAD) and group C (10^2^ HAD).

Experimental infection day was defined as day 0. After inoculation, clinical signs and rectal temperatures were monitored daily. Blood samples were taken prior to inoculation, then at 2- or 3-day intervals post inoculation into EDTA vacutainers (BD biosciences, Eysins, Switzerland). Viral nucleic acid was extracted from blood samples using Viral RNA mini kit (Qiagen, Manchester, UK) and viral DNA levels (genome copies) were determined by real time PCR.

### 4.2. Evaluation of Lesions and Detection of African Swine Fever Virus in Tissue Samples

During necropsies, macroscopic lesions were evaluated following a revised version of previous protocols [37] which were adapted to include simplified pathological evaluation criteria and agreed by veterinary pathologists from different institutions participating in the ASF-Stop Cost Action Consortium (See Supplementary Material S1 (evaluation criteria of macroscopic lesions and scoring system) and Supplementary Material S2 (macroscopic lesions scoring sheet).

A huge range of tissue samples (Supplementary Material S3) were taken, fixed for 7 days in 10% buffered formalin solution and routinely processed for histopathological and immunohistochemical studies. Histopathological lesions were evaluated following pathological evaluation criteria in combination with a semi-quantitative scoring system as follows: (0) no lesion; (1) minimal lesion; (2) mild lesion; (3) moderate lesion; (4) severe lesion (Supplementary Material S3: histopathological evaluation criteria and scoring system).

Tissue sections were also used for immunohistochemical demonstration of ASFV by a monoclonal antibody against the virus protein p30/CP204L (The Pirbright Institute, Pirbright, UK). Briefly, sections were deparaffinised and rehydrated and endogenous peroxidase activity was quenched by incubation with 3% hydrogen peroxide in methanol for 15 min (min) at room temperature (RT). Then, samples were subjected to heat-induced epitope retrieval by immersion in DAKO buffer (pH 9) at 100 °C for 10 min. After treatment, sections were cooled at RT (20 min) and rinsed in TBST buffer wash (pH 7.2) for 5 min. Nonspecific binding sites were blocked with normal serum block (5% normal goat, 5% normal swine, 5% normal chicken in TBST buffer) for 20 min. After the blocking step, sections were incubated with a primary monoclonal antibody against virus protein p30/CP204L (10 µg/mL in TBST buffer) for 1 h at RT. After primary incubation, sections were washed with TBST buffer (2 × 5 min). EnVision (DAKO, Glostrup, Denmark) was utilized for visualization (30 min. incubation at RT) and 3,3′-diaminobenzidine (DAB) 0.05% solution in Mcllvaine’s phosphate citrate buffer containing hydrogen peroxide 0.001% was applied for 10 min. as the chromogen. Then, samples were rinsed with distilled (1 × 5 min) and tap water (1 × 5 min). Finally, sections were counterstained with Mayer’s haematoxylin, dehydrated and mounted. Tissue samples from infected and noninfected pigs were included as test controls. In addition, specific primary antibody was replaced by (1) PBS and (2) a subtype matching IgG to serve as two primary antibody-omit negative technique controls. A detailed protocol including all steps and reagents used is provided (Supplementary Material S4: ASFV detection by immunohistochemistry).

A semiquantitative assessment of cells immunolabeled for viral antigen (protein p30/CP204L) was performed on different histological structures of organs which were evaluated as follows: (0) no presence of immunolabeled cells; (1) occasional presence of immunolabeled cells; (2) mild presence of immunolabeled cells; (3) moderate presence of immunolabeled cells; (4) abundant presence of immunolabeled cells. Morphological features, location and size were the criteria applied to identify the types of immunolabeled cells (Supplementary Material S5: scoring of cells immunolabeled for viral antigen in different histological structures; Supplementary Figure S1: examples for the assessment of cells immunolabeled for viral antigen in different organs).

### 4.3. Statistical Analysis

Macroscopic and histopathological scores along with the scores of cells immunolabeled for viral antigen were expressed as medians with 95% confidence interval (CI). Differences among experimental groups were tested for statistical significance using one-way ANOVA. All statistical analyses and data visualisation were performed with GraphPad Prism Version 7.0 (GraphPad Software, La Jolla, CA, USA).

## 5. Conclusions

In this study, we made use of an intranasal infection model for ASFV where the dose of infection influenced the outcome of clinical presentation, and where the correlation of clinical scores with pathological lesions (both gross- and histopathology) was reviewed. The study corroborates the moderate virulence of the Ken05/Tk1 isolate, as well as its capacity to induce both acute and, occasionally, subacute forms of ASF when medium and high doses were administered intranasally. The severity of vascular lesions induced by high and medium doses was not associated with the amount of virus detected in tissues and might be attributed to indirect mechanisms not evaluated in the present study. The proposed evaluation protocols enable the simplification of the pathological evaluations carried out in ASF studies and the set-up differences among animals and experimental groups in a faster, more accurate and reliable way, also facilitating the standardized and harmonized comparison of ASF lesions across studies.

## Figures and Tables

**Table 1:** Detection of viraemia, incubation period and clinical course in domestic pigs inoculated by intranasal route with different doses of ASF isolate Ken05/Tk1 (Genotype X).

Pig No.	Viraemia *	Incubation Period	Clinical Course	Euthanasia
Group A—High dose (10^4^ HAD_50_)				
86	5 pi (low levels)	7	5	11 pi
87	5 pi (high levels)	5	5	9 pi
88	10 pi (low levels)	NCS	NCS	12 pi **
89	5 pi (high levels)	6	7	12 pi
90	5 pi (high levels)	5	5	9 pi
Group B—Medium dose (10^3^ HAD_50_)				
81	10 pi (moderate levels)	7	14	20 pi **
82	13 pi (high levels)	7	7	13 pi
83	12 pi (low levels)	17	4	20 pi
84	12 pi (low levels)	15	4	18 pi
85	12 pi (low levels)	13	4	16 pi

* First day in which viraemia was detected after infection (pi) with Ken05/Tk1 isolate. Duration of incubation period and clinical course are given in days. ** Did not reach clinical end point; Euthanised to prevent single housing. NCS: not observed clinical signs.

## Data Availability

Not applicable.

## Supplementary Materials

The following are available online at https://www.mdpi.com/article/10.3390/pathogens10060768/s1; File S1: evaluation criteria of macroscopic lesions and scoring system; File S2: macroscopic lesions scoring sheet; File S3: histopathological evaluation criteria and scoring system; File S4: ASFV detection by immunohistochemistry; File S5: scoring of cells immunolabeled for viral antigen in different histological structures; Figure S1: examples for the assessment of cells immunolabeled for viral antigen in different organs.
