# Supplementary material for: Evaluation of Lesions and Viral Antigen Distribution in Domestic Pigs Inoculated Intranasally with African Swine Fever Virus Ken05/Tk1 (Genotype X)
Source: Pathogens. 2021 Jun 18;10(6):768. doi: 10.3390/pathogens10060768 (PMC8234863; doi:10.3390/pathogens10060768)
Supplement: Supplementary file 1 [file pathogens-10-00768-s001.zip › pathogens-1253033-supplementary/Suppl. Figure 1.pdf]

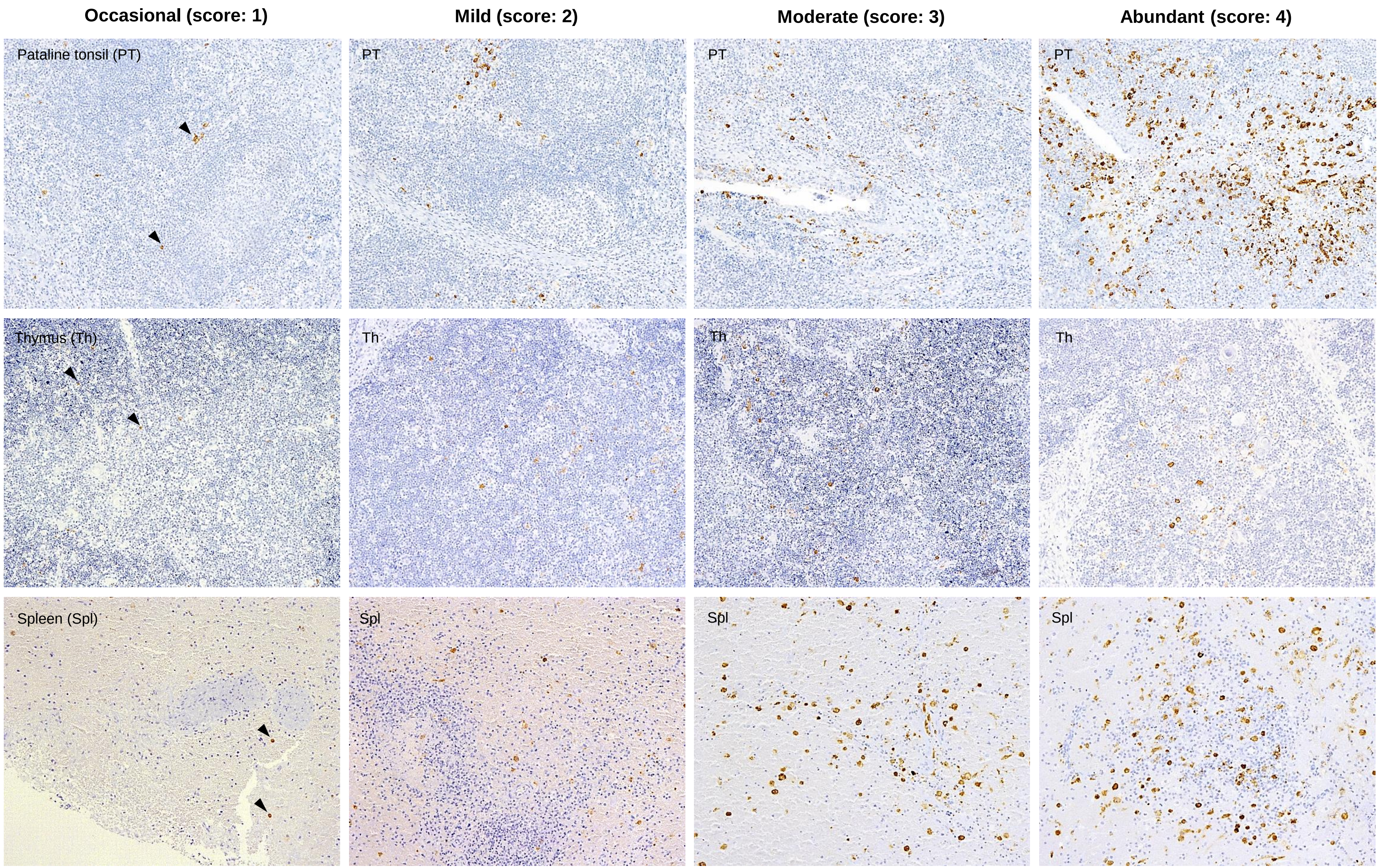
Occasional (score: 1)
Mild (score: 2)
Moderate (score: 3)
Abundant (score: 4)
Pataline tonsil (PT)
PT
PT
PT
Thymus (Th)
Th
Th
Th
Spleen (Spl)
Spl
Spl
Spl

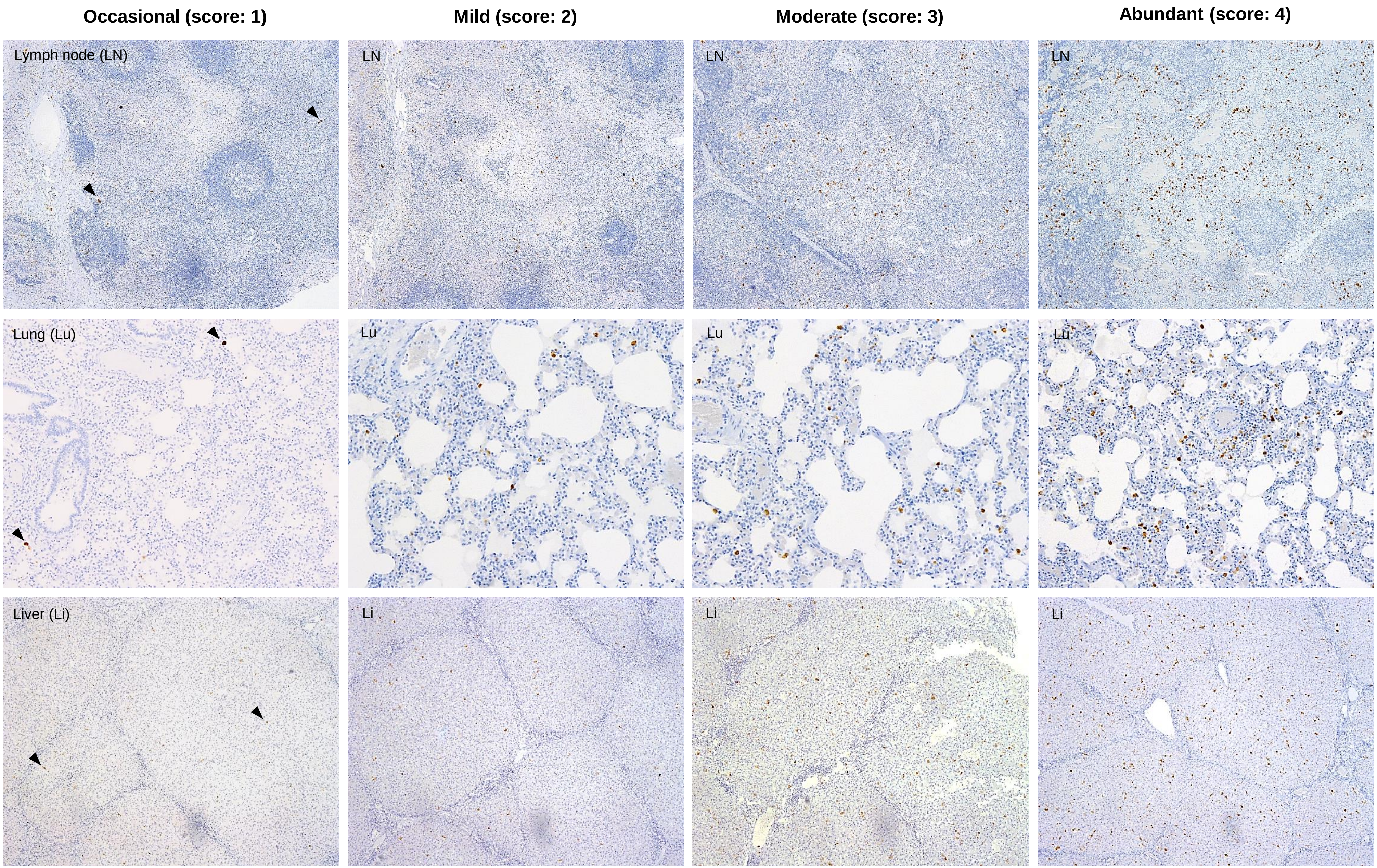

Occasional (score: 1)
Mild (score: 2)
Moderate (score: 3)
Abundant (score: 4)
Lymph node (LN)
LN
LN
LN
Lung (Lu)
Lu
Lu
Lu
Liver (Li)
Li
Li
Li
