# Supplementary material for: Evaluation of Lesions and Viral Antigen Distribution in Domestic Pigs Inoculated Intranasally with African Swine Fever Virus Ken05/Tk1 (Genotype X)
Source: Pathogens. 2021 Jun 18;10(6):768. doi: 10.3390/pathogens10060768 (PMC8234863; doi:10.3390/pathogens10060768)
Supplement: Supplementary file 1 [file pathogens-10-00768-s001.zip › pathogens-1253033-supplementary/Supplementary material 1. Evaluation criteria of macroscopic lesions and scoring system.pdf]

Organ or System	Score	Description/Lesions
**External examination**		
Body condition	0-3	(1) Vertebrae, ribs and bony protuberances only detectable with firm pressure.<br>(2) Vertebrae, ribs and bony protuberances prominent.<br>(3) Vertebrae, ribs and bony protuberances very prominent.
Eyes/ocular mucosa	0-3	(1) Mild hyperaemia.<br>(2) Moderate hyperaemia, swelling of eyelids and clear ocular discharge.<br>(3) Congested ocular mucosa with haemorrhages, inflamed eyelids and severe cloudy ocular discharge (gummed up eyes).
Nose/nasal cavity	0-3	(1) Mild seromucous nasal discharge.<br>(2) Moderate mucous nasal discharge.<br>(3) Severe mucous nasal discharge.
		(3) Foamy material in nostrils.
		(3) Nasal haemorrhages (epistaxis)/blood traces around nostrils.
Oral cavity	0-3	(3): Foamy material in mouth.
		(3) Necrotic areas in tongue.
Skin	0-3	(1) Small or (2) moderate-size areas with skin erythema (ears, snout, periorbital region, neck, ventral chest, dorsal-lateral-caudal skin areas, abdomen, scrotal sac, perianal area, tail, distal limbs).<br>(3) Generalized reddening of the skin with haemorrhages all over the body.
		(1) Small skin areas with mild cyanotic changes or (2) moderate-size skin areas with moderate cyanotic changes (ears, snout, periorbital region, neck, ventral chest, dorsal-lateral-caudal skin areas, abdomen, scrotal sac, perianal area, tail, distal limbs).<br>(3) Large skin areas with severe cyanotic changes.
		(1) Occasional presence of small necrotic areas on skin or small raised necrotic skin foci.<br>***(2) Presence of multifocal moderate-size necrotic areas on skin or multifocal moderate-size raised necrotic skin foci.***<br>***(3) Presence of large skin areas with severe necrotic areas /chronic ulcers all over the body.***
		(3) Varioliform skin eruptions.
		(3) Abscess.
Subcutaneous areas	0-3	(1) Occasional presence of small areas with mild subcutaneous oedema.<br>(2) Multifocal moderate-size areas with mild to moderate subcutaneous oedema, small haemorrhages and haematomas.<br>(3) Multifocal large-size areas with moderate to severe subcutaneous oedema, moderate to severe haemorrhages and haematomas.
**Musculoskeletal system**		
Skeletal muscle	0-3	(1) Small areas with mild oedema among the fascial planes.<br>(2) Multifocal moderate-size areas with mild to moderate oedema and small haemorrhages among the fascial planes.<br>(3) Multifocal large-size areas with moderate to severe oedema and moderate to severe haemorrhages among the fascial planes.
		(3) Pale areas/skeletal muscle necrosis.
Joints	0-3	***(1) Mild or (2) moderate joint swelling with serofibrinous/serohaemorrhagic periarthritis/arthritis with moderate/abundant red-tinged fluid.***<br>***(3) Severe joint swelling with purulent periarthritis/arthritis.***
**Thoracic cavity/Cardiorespiratory system**		
Exudates	0-3	(1) Mild, (2) moderate or (3) severe hydrothorax with yellowish or red-tinged fluid.
		(3) Haemothorax.
Pericardium/pericardial sac	0-3	(1) Mild, (2) moderate or (3) severe hydropericardium with yellowish or red-tinged fluid.
		(3) Haemopericardium.
		(3) Fibrinous pericarditis
Heart	0-3	(1) Petechial haemorrhages on epicardium.<br>(2) Variable presence of petechial haemorrhages/ecchymoses on epicardium, myocardium and endocardium.<br>(3) Haemorrhagic heart with extensive coalescent haemorrhages (ecchymoses) on epicardium, myocardium and endocardium.
Lung (lack of collapse)	0-3	(1) Mild lack of collapse with no ribs impressions.<br>(2) Moderate lack of collapse with mild or scarce ribs impressions.<br>(3) Moderate to severe lack of collapse with apparent ribs impressions.

Lung (congestion/haemorrhages)	0-3	(1) Mild congestion or active hyperaemia, diffuse or patchy distributed in parenchyma. No haemorrhages.<br>(2) Variable degree of congestion or active hyperaemia. Multifocal to coalescent randomly distributed petechiae and purpurae.<br>(3) Variable degree of congestion/hyperaemia. Multifocal to coalescent random and interlobular distributed haemorrhages (ecchymoses).
Alveolar/interstitial oedema	0-3	(1) Scarce or no presence of foamy material in trachea/bronchus (alveolar oedema) and minimal distension of interlobular walls (interstitial oedema).<br>(2) Mild to moderate presence of foamy material in trachea/bronchus and moderate distension of interlobular walls.<br>(3) Marked presence of foamy material in trachea/brochus and severe distension of interlobular walls.
Consolidation of pulmonary parenchyma	0-3	(1) Small cranio-ventral (uni/bilateral) areas of consolidation.<br>(2) Moderate-size cranio-ventral (uni/bilateral) areas of consolidation.<br>(3) Large cranio-ventral (uni/bilateral) areas of consolidation with red and grey hepatization of lobules.
		(3) Necrotic pneumonia.
		(3) Purulent lesions.
Pleura	0-3	***(3) Fibrinous pleuritis/pleural adhesions.***
**Abdominal cavity/Gastrointestinal system**		
Ascites	0-3	(1) Mild, (2) moderate or (3) severe ascites with yellowish or red-tinged fluid.
		(3) Haemoabdomen.
Stomach contents	0-3	(1) Absence of gastric content.<br>(2) Scarce mucous-liquid content.<br>(3) Presence of digested blood content (black) or fresh hemorrhagic content (red).
Stomach	0-3	(1) Petechiae in serosal surface.<br>(2) Petechiae and ecchymoses in serosal surface.<br>(3) Hyperemia/congestion, petechiae, ecchymoses and suggillations in serosal and mucosal surfaces along with clotted blood.
		(3) Necrotic areas/ulcers in mucosal surface.
Duodenum	0-3	(1) Petechiae in serosal surface.<br>(2) Petechiae and ecchymoses in serosal surface.<br>(3) Hyperemia/congestion, petechiae, ecchymoses and suggillations in serosal and mucosal surfaces along with clotted blood.
Jejunum	0-3	
Ileum	0-3	***(3) Necrotic areas/ulcers in mucosal surface.***
Ileocecal valve	0-3	
Cecum	0-3	
Colon	0-3	
Rectum	0-3	
Liver	0-3	(1) Mild multifocal parenchymatous colour changes with slight hepatomegaly.<br>(2) Hepatomegaly, moderate congestion and presence multifocal punctiform redness areas/subcapsular haemorrhages (petechiae). Moderate bleeding after sectioning.<br>(3) Hepatomegaly, severe congestion and presence of coalescent punctiform redness areas/ subcapsular haemorrhages (petechiae and purpurae). Marked bleeding after sectioning.
Gallbladder/bile duct	0-3	(1) Mild to moderate oedema affecting gallbladder wall/bile duct.<br>(2) Moderate oedema affecting gallbladder wall/bile duct with haemorrhages on serosal/submucosal surface.<br>(3) Severe oedema affecting gallbladder wall/cystic duct with severe haemorrhages on serosal/submucosal surface.
**Urinary system**		
Kidney (perirenal oedema)	0-3	(1) Mild, (2) moderate or (3) severe perirenal oedema
Parenchymal kidney haemorrhages	0-3	(1) Occasional presence of multifocal petechiae in the renal cortex and medulla with scarce colour changes.<br>(2) Abundant presence of multifocal haemorrhages (petechiae/ecchymoses) in the cortex, medulla and renal pelvis along with moderate pelvic dilation and diffuse mild to moderate congestion.<br>(3) Severe diffuse haemorrhages (petechiae, ecchymoses and purpura) in the capsule, cortex, medulla and renal pelvis along with marked pelvic dilation and diffuse severe congestion (generalized renal darkening).
Urinary bladder	0-3	(1) Mild multifocal petechiae in sumucosa without/with mild colour changes.<br>(2) Multifocal petechiae and ecchymoses in submucosa and serosa with mild to moderate oedema affecting urinary bladder wall. Mild to moderate hyperemia/congestion on mucosal surface.

		(3) Coalescent severe haemorrhages (petechiae, ecchymoses and suggillations) in submucosa and serosa along with blood clots covering large areas of the mucosal surface, severe oedema affecting urinary bladder wall and severe hyperemia/congestion on mucosal surface.
**Lymphoid system**		
Palatine tonsils	0-3	(1) Mild erythema, (2) moderate eythema or (3) severe erythema with petechial haemorrhages in tonsils.
		(3) Necrotic areas in tonsils.
Thymus	0-3	(1) Occasional petechiae.<br>(2) Moderate colour changes with occasional petechiae.<br>(3) Moderate colour changes with petechiae and ecchymoses.
		(1) Mild, (2) moderate or (3) severe thymus atrophy (in young animals)
Spleen	0-3	(1): Mild hyperemic splenomegaly (almost normal size and colour close to normal, not friable consistency displaying minimal to mild bleeding after sectioning).<br>(2): Moderate/partial hyperemic splenomegaly (moderately enlarged displaying moderate bleeding after sectioning)<br>(3): Severe hyperemic splenomegaly (enlarged several times its normal size, rounded edges, friable consistency and dark red to black colour, marked bleeding after sectioning).
		(3) Focal necrosis/infarcts.
		(3) Hyperplasic splenomegaly (rubbery in consistency, no bleeding after sectioning)/cicatrization of partial hyperemic splenomegaly).
Lymph nodes (LNs):		(1): Mild increase in size, oedematous with mild colour changes.<br>(2): Moderate increase in size, oedematous and partially haemorrhagic (petechiae and ecchymoses).<br>(3): Markedly enlarged, edematous aspect and completely haemorrhagic with an appearance similar to a blood clot.
• Superficial inguinal LNs	0-3	
• Parotid LNs	0-3	
• Pre-scapular LNs	0-3	
• Submandibular LNs	0-3	
• Lateral retropharyngeal LNs	0-3	
• Medial retropharyngeal LNs	0-3	
• Mediastinal LNs	0-3	***(3) Hyperplasia without haemorrhagic changes or with occasional peripheral haemorrhages (lymph nodes appear enlarged, diffusely white or tan, and rubbery in consistency).***
• Tracheobronchial LNs	0-3	
• Gastrohepatic LNs	0-3	
• Renal LNs	0-3	
• Mesenteric LNs	0-3	
• Ileocaecal LNs	0-3	
• Other LNs	0-3	
**Endocrine system**		
Thyroid gland	0-1	(1): Increased size/haemorrhages.
Adrenal gland	0-1	
Pancreas	0-1	
**Central nervous system**		
Telencephalon	0-3	(1): Hyperemia in meninges/oedema.<br>(2): Hyperemia in meninges/oedema with occasional petechiae.<br>(3): Severe vascular changes with oedema, frequent petechiae and ecchymoses.
Cerebellum	0-3	(3): Meningitis (meninges appear thickened with loss of transparency)
Medulla oblongata	0-3	

(Bold italics): *Lesions induced by low virulent ASFV isolates or more commonly described in ASF chronic forms. Opportunistic pathogens, mainly bacteria, are considered as the ultimately responsible for the appearance of some of these macroscopic lesions.*
