# Supplementary material for: Evaluation of Lesions and Viral Antigen Distribution in Domestic Pigs Inoculated Intranasally with African Swine Fever Virus Ken05/Tk1 (Genotype X)
Source: Pathogens. 2021 Jun 18;10(6):768. doi: 10.3390/pathogens10060768 (PMC8234863; doi:10.3390/pathogens10060768)
Supplement: Supplementary file 1 [file pathogens-10-00768-s001.zip › pathogens-1253033-supplementary/Supplementary material 3. Histopathological evaluation criteria and scoring system.pdf]

**Histopathologic lesions**	**Pig No.**	**Pig No.**	**Pig No.**	**Pig No.**	**Observations/notes**
**Lung** (pulmonary lobe:) -Hyperaemia/angiectasia					
-Haemorrhages (perivascular/peribronchial/alveolar)					
-Vasculopathy*/microthrombosis					
-Interstitial oedema					
-Alveolar oedema					
-Thickening of alveolar septal					
-Cells in alveolar lumen					
-Cell debris/fibrin in alveolar lumen					
-Cell death/cellular fragmentation in BALT/TBMs					
-Bronchi and bronchioles with cell debris, fibrin and detached epithelium/inflammatory cells					
-Peribronchial/bronchiolar mononuclear infiltrates					
**Pleura** -Pleural thickening					
-Fibrinous pleuritis					

*Vasculopathy: prominent diffuse endothelial activation (cellular hypertrophy, rounded nuclei) and variable smooth myocyte vacuolation of tunica media of small and medium vessels, with (vasculitis)/without intramural inflammatory infiltration.
(BALT): bronchus-associated lymphoid tissue; (TBMs): tingible body macrophages.

Histopathological scoring system: (0) No lesion; **(1)** Minimal lesion; **(2)** Mild lesion; **(3)** Moderate lesion; **(4)** Severe lesion.

Histopathologic lesions	Pig No.	Pig No.	Pig No.	Pig No.	Observations/notes
**Distal ileum**<br>-Shortened/thickened mucosal villi					
-Mononuclear infiltrate in lamina propria					
-Hyperaemia/haemorrhages (submucosa/serosa)					
-Lymphoid depletion of Peyer´s patches/ IFA					
-Cellular fragmentation/TBMs					
-Vasculopathy*					
**Ileocaecal valve**<br>-Mononuclear infiltrate in lamina propria					
-Hyperaemia/haemorrhages (submucosa/serosa)					
-Lymphoid depletion of lymphoid follicles/IFA					
-Cellular fragmentation/TBMs					
-Vasculopathy*					
**Colon**<br>-Hyperaemia/haemorrhages (epithelium)					
-Mononuclear infiltrate (lamina propria)					
-Hyperaemia/haemorrhages/ oedema (submucosa)					
-Lymphoid depletion of lymphoid follicles					
-Cellular fragmentation/TBMs					
-Vasculopathy*					

*Vasculopathy: prominent diffuse endothelial (EC) activation (cellular hypertrophy, rounded nuclei) and variable smooth myocyte vacuolation of tunica media of small and medium vessels with (vasculitis)/without intramural inflammatory infiltration; (IFA): interfollicular area; (TBMs): tingible body macrophages.

Histopathological scoring system: (0) No lesion; **(1)** Minimal lesion; **(2)** Mild lesion; **(3)** Moderate lesion; **(4)** Severe lesion.

**Histopathologic lesions**	**Pig No.**	**Pig No.**	**Pig No.**	**Pig No.**	**Observations/notes**
**Kidney**<br>-Congestion<br>(cortex/medulla/renal pelvis)					
-Haemorrhages (cortex/medulla/renal pelvis)					
-Interstitial nephritis/cellular fragmentation					
-Membranoproliferative glomerulonephritis					
-Tubulonephrosis<br>(intratubular hyaline droplets/casts)					
-Vasculopathy*/Microthrombosis					

*Vasculopathy: prominent diffuse endothelial cell (EC) activation (cellular hypertrophy, rounded nuclei) and variable smooth myocyte vacuolation of tunica media of small and medium vessels with (vasculitis)/without intramural inflammatory infiltration.

Histopathological scoring system: (0) No lesion; **(1)** Minimal lesion; **(2)** Mild lesion; **(3)** Moderate lesion; **(4)** Severe lesion.

**Histopathologic lesions**	**Pig No.**	**Pig No.**	**Pig No.**	**Pig No.**	**Observations/notes**
**Spleen**<br>-Engorgement of red pulp sinuses					
-Follicular lymphoid depletion					
-Periarteriolar lymphoid sheaths (PLS) depletion					
Cellular fragmentation/TBMs in LF/PLS					
-Haemorrhages within white pulp structures					
-Vascular damage*/fibrin deposits					
-Ellipsoids increased in size/depletion					
**Thymus**<br>-Congestion/haemorrhages (cortex/medulla)					
-Cortex atrophy (cortex)					
-Lymphoid depletion (medulla)					
Cellular fragmentation/TBMs in cortex/medulla					
-Vasculitis*/perivasculitis					
**Tonsil**<br>-Hyperaemia / Haemorrhages					
-Mononuclear infiltrates (epithelium of the crypts)					
-Follicular lymphoid depletion					
-Depletion in interfollicular areas (IFA)					
Cellular fragmentation/TBMs in LF/IFA					
-Vasculopathy*					
-Connective tissue proliferation (IFA)					

*Vasculopathy: prominent diffuse endothelial cell (EC) activation (cellular hypertrophy, rounded nuclei) and variable smooth myocyte vacuolation of tunica media of small and medium vessels with (vasculitis)/without intramural inflammatory infiltration; (LF): lymphoid follicles; (IFA) interfollicular areas; (PLS): periarterial lymphoid sheaths; (TBMs): tingible body macrophages.

Histopathological scoring system: (0) No lesion; **(1)** Minimal lesion; **(2)** Mild lesion; **(3)** Moderate lesion; **(4)** Severe lesion.

**Histopathologic lesions**	**Pig No.**	**Pig No.**	**Pig No.**	**Pig No.**	**Observations/notes**
**Lymph nodes**<br>LN identification:<br>-Hyperaemia/congestion					
-Haemorrhages					
-Follicullar lymphoid depletion					
-Interfollicular area depletion					
-Cellular fragmentation/TBMs in LF/IFA.					
-Vascular damage*/fibrin deposits					
**Bone marrow**<br>-Hypocellularity					
-Cell debris					
-Fibrin strands					
-Microhaemorrhages					
-MKs (relative number)					
-Presence of apoptotic MKs					
-Presence of cloud-nuclei MKs/Clusters					
-Emperipolesis					

*(Vasculopathy): prominent diffuse endothelial cell (EC) activation (cellular hypertrophy, rounded nuclei) and variable smooth myocyte vacuolation of tunica media of small and medium vessels with (vasculitis)/without intramural inflammatory infiltrations.
(MK): megakaryocytes; (LF): lymphoid follicles; (IFA): interfollicular areas; (TBMs) tingible body macrophages.
(Cloud-nuclei MKs): megakaryocytes with elongated cytoplasm and a round, balloon-shaped nucleus located at one end of the cell.
(Apoptotic MKs): megakaryocytes with condensed, compact hyperchromatic nucleus; cytoplasm was either absent or formed a small perinuclear halo.
(Emperipolesis): morphological normal neutrophils, embedded in MK cytoplasm.

Histopathological scoring system: **(0)** No lesion; **(1)** Minimal lesion; **(2)** Mild lesion; **(3)** Moderate lesion; **(4)** Severe lesion.

Histopathologic lesions	Pig No.	Pig No.	Pig No.	Pig No.	Observations/notes
**Skin**<br>**Epidermis**					
-Subcorneal/intraepithelial pustules					
-Inflammatory mononuclear infiltrates					
-Necrotizing ulcerative dermatitis					
**Dermis**					
-Folliculitis					
-Intravascular monocytes/neutrophils					
-Perivascular mononuclear infiltrate/apoptosis					
-Perivascular oedema					
-Inflammatory interstitial infiltrates/apoptosis					
-Vasculitis*, necrosis, fibrin in blood vessels					
-Hyperemia					
-Microhaemorrhages					
**Liver**<br>-Angiectasia (PT/CV)/hepatic sinusoids expanded					
-Haemorrhages.					
-Vasculopathy*/ Microthrombosis					
-Interstitial mononuclear infiltrates					
-Interstitial cellular fragmentation					
-Necrotic foci in lobules					
-Enlarged Kupffer cells in hepatic sinusoids					
Increase of circulating leukocytes (sinusoidal leucocytosis)					
-Hypertrophy/hyperplasia of bile ducts					

*(Vasculopathy): prominent diffuse endothelial (EC) activation (cellular hypertrophy, rounded nuclei) and variable smooth myocyte vacuolation of tunica media of small and medium vessels with (vasculitis)/without intramural inflammatory infiltrations. (PT): portal triad; (CV): central vein

Histopathologic lesions: (0) No lesion; **(1)** Minimal lesion; **(2)** Mild lesion; **(3)** Moderate lesion; **(4)** Severe lesion.
