# Supplementary material for: Evaluation of Lesions and Viral Antigen Distribution in Domestic Pigs Inoculated Intranasally with African Swine Fever Virus Ken05/Tk1 (Genotype X)
Source: Pathogens. 2021 Jun 18;10(6):768. doi: 10.3390/pathogens10060768 (PMC8234863; doi:10.3390/pathogens10060768)
Supplement: Supplementary file 1 [file pathogens-10-00768-s001.zip › pathogens-1253033-supplementary/Supplementary material 5. Scoring of cells immunolabeled against viral antigen.pdf]

**Positive cells (IHC-p30/CP204L)**	**Pig No.**	**Pig No.**	**Pig No.**	**Pig No.**	**Observations/notes**
**Lung** (pulmonary lobe:)*					
-PIMs/Interstitial Mos					
-PAMs					
-BALT					
-Pneumocytes					
-Other cells/areas					
**Lung** (pulmonary lobe:)*					
-PIMs/Interstitial Mos					
-PAMs					
-BALT					
-Pneumocytes					
-Other cells/areas					

*The morphological identification of cells immunolabeled against viral antigen in each of the histological areas/structures evaluated must be also included.
(Mos): macrophages.
(PIM): pulmonary intravascular macrophages.
(PAM): pulmonary alveolar macrophages.
(BALT): bronchus-associated lymphoid tissue.

Scoring of cells immunolabeled for viral antigen: (0) no presence of immunolabeled cells; **(1)** occasional presence of immunolabeled cells; **(2)** mild presence of immunolabeled cells; **(3)** moderate presence of immunolabeled cells; **(4)** abundant presence of immunolabeled cells.

**Positive cells (IHC-p30/CP204L)**	**Pig No.**	**Pig No.**	**Pig No.**	**Pig No.**	**Observations/notes**
**Distal ileum*** -Epithelium					
-Lamina propria					
-Lymphoid follicles (LF)					
-Interfollicular areas					
-Other cells/areas					
**Ileocaecal valve*** -Epithelium					
-Lamina propria					
-Lymphoid follicles (LF)					
-Interfollicular areas					
-Other cells/areas					
**Colon*** -Epithelium					
-Lamina propia					
-Submucosa (occasional LF)					
-Other cells/areas					
**Kidney*** -Glomeruli (cortex)					
-Interstitial infiltrates (cortex)					
-Medulla					
-Renal pelvis					
-Other cells/areas					

*The morphological identification of cells immunolabeled against viral antigen in each of the histological areas/structures evaluated must be also included.

Scoring of cells immunolabeled for viral antigen: **(0)** no presence of immunolabeled cells; **(1)** occasional presence of immunolabeled cells; **(2)** mild presence of immunolabeled cells; **(3)** moderate presence of immunolabeled cells; **(4)** abundant presence of immunolabeled cells.

**Positive cells (IHC-p30/CP204L)**	**Pig No.**	**Pig No.**	**Pig No.**	**Pig No.**	**Observations/notes**
**Spleen*** -Red pulp					
-Lymphoid follicles					
-Periarteriolar lymphoid sheaths					
-Ellipsoids					
-Other cells/areas					
**Thymus*** -Cortex					
-Corticomedullary junction.					
-Medulla					
-Other cells/areas					
**Tonsil*** -Epithelium (crypts)/lamina. propria					
-Lymphoid follicles					
-Interfollicular areas					
-Other cells/areas					

*The morphological identification of cells immunolabeled against viral antigen in each of the histological areas/structures evaluated must be also included.

Scoring of cells immunolabeled for viral antigen: (0) no presence of immunolabeled cells; **(1)** occasional presence of immunolabeled cells; **(2)** mild presence of immunolabeled cells; **(3)** moderate presence of immunolabeled cells; **(4)** abundant presence of immunolabeled cells.

Positive cells (IHC-p30/CP204L)	Pig No.	Pig No.	Pig No.	Pig No.	Observations/notes
**Lymph nodes*** **LN identification:**					
-Lymphoid follicles					
-Interfollicular areas					
-Medulla					
-Other cells/area					
**LN identification:***					
-Lymphoid follicles					
-Interfollicular areas					
-Medulla					
-Other cells/area					
**LN identification:***					
-Lymphoid follicles					
-Interfollicular areas					
-Medulla					
-Other cells/area					
**LN identification:***					
-Lymphoid follicles					
-Interfollicular areas					
-Medulla					
-Other cells/area					
**LN identification:***					
-Lymphoid follicles					
-Interfollicular areas					
-Medulla					
-Other cells/area					
**LN identification:***					
-Lymphoid follicles					
-Interfollicular areas					
-Medulla					
-Other cells/area					

*The morphological identification of cells immunolabeled against viral antigen in each of the histological areas/structures evaluated must be also included.

Scoring of cells immunolabeled for viral antigen: (0) no presence of immunolabeled cells; **(1)** occasional presence of immunolabeled cells; **(2)** mild presence of immunolabeled cells; **(3)** moderate presence of immunolabeled cells; **(4)** abundant presence of immunolabeled cells.

**Positive cells (IHC-p30/CP204L)**	**Pig No.**	**Pig No.**	**Pig No.**	**Pig No.**	**Observations/notes**
**Bone marrow**					
-Granulocytic cells					
-Megakaryocytes					
-Reticular/myeloid cells					
-Other cells/area					
**Skin**<br>**Epidermis**					
-Epithelium					
**Dermis**					
-Intravascular monocytes/neutrophils					
-Perivascular mononuclear infiltrate					
-Endothelial cells (capillaries)					
-Reticular cells					
-Other cells/area					
**Liver**<br>-Circulating monocytes					
-Kupffer's cells					
-Interstitial macrophages					
-Hepatocytes					
-Other cells/area					

*The morphological identification of cells immunolabeled against viral antigen in each of the histological areas/structures evaluated must be also included.

Scoring of cells immunolabeled for viral antigen: (0) no presence of immunolabeled cells; **(1)** occasional presence of immunolabeled cells; **(2)** mild presence of immunolabeled cells; **(3)** moderate presence of immunolabeled cells; **(4)** abundant presence of immunolabeled cells.
